# Cell‐in‐Bead‐in‐Droplet Platform for pH‐Based Microfluidic Screening of Ureolytic Bacteria

**DOI:** 10.1002/smll.202508107

**Published:** 2026-02-18

**Authors:** Diego Giovanoli, Nadia Enrriquez, Anton Kan, Mathias Steinacher, Raphael Buess, Tomas Pena Müller, Stavros Stavrakis, Andrew deMello, André R. Studart

**Affiliations:** ^1^ Complex Materials Department of Materials ETH Zürich Zürich Switzerland; ^2^ Institute For Chemical and Bioengineering Department of Chemistry and Applied Biosciences ETH Zürich Zurich Switzerland

**Keywords:** bioprospection, evolution, high‐throughput sorting, microbially induced calcium carbonate precipitation, microorganisms

## Abstract

Ureolytic microorganisms are central to microbially induced carbonate precipitation (MICP), a biotechnological process with applications in construction, environmental remediation, and wastewater treatment. Despite their potential, the discovery of robust, high‐performing ureolytic strains is limited by the lack of assays that measure single‐cell enzymatic activity in high‐throughput platforms, such as droplet microfluidic devices. Although pH‐based assays using urea offer a direct and label‐free readout of urease activity, their implementation in droplet microfluidics is hindered by chemical crosstalk through diffusing molecules. Ammonia, the volatile product of ureolysis, spreads between droplets, making it difficult to detect droplets that host high‐performing cells. To overcome this limitation, we have developed a ‘cell‐in‐bead‐in‐droplet’ (CiBiD) microfluidic platform that enables reliable detection of localized pH changes within individual cell‐laden droplets. Single bacterial cells are first encapsulated in agarose beads to proliferate into microcolonies. The cell‐laden beads are then re‐encapsulated into droplets containing urea, a pH‐sensitive fluorescent dye, and a buffer. By boosting the local enzymatic activity in the droplet while neutralizing diffusing ammonia with the buffer, the CiBiD approach circumvents diffusional crosstalk and enables robust detection of urease activity based on localized pH variations. Using a mock microbial consortium, our system achieved a 25‐fold enrichment of active ureolytic strains after sorting 628 out of approximately 240,000 droplets in less than 30 min. To demonstrate its potential for the biopropection of functional microorganisms from natural microbiomes, the methodology was also successfully utilized to enrich ureolytic bacteria from environmental soil samples. Beyond local pH detection, the CiBiD concept may be applied to other challenging assays in media that operate at extreme pHs, involve high salt concentrations or are prone to undesirable dye interference. This makes CiBiD an attractive screening tool for high‐throughput bioprospection and directed evolution of microorganisms.

## Graphical abstract

Efficient high‐throughput microbial screening in droplet microfluidics is often challenged by chemical crosstalk between droplets. This is particularly difficult in pH‐changing biological processes, for which more effective biochemical assays are needed. The Cell‐in‐Bead‐in‐Droplet (CiBiD) concept overcomes this limitation by decoupling microbial growth and activity detection. This enables effective screening of ureolytic microorganisms from complex libraries or soil microbiomes.

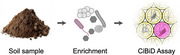

## 1 Introduction

Microorganisms and their enzymes drive innovation in biocatalysis, drug discovery, bioremediation, and synthetic biology. The ability of microorganisms to catalyze complex reactions with high specificity under mild conditions makes them attractive alternatives to traditional chemical processes. Although microbes offer immense technological potential, fewer than one percent of species have been cultivated, and their ecological and functional traits remain largely unknown [1, 2]. Among microbial traits of technological relevance, ureolysis is of particular interest due to its role in microbially induced carbonate precipitation (MICP). MICP is a process relevant for sustainable technologies such as self‐healing concrete [3, 4], wastewater treatment [5, 6], and soil stabilization [7, 8]. In this process, ureolytic bacteria hydrolyze urea into ammonia and carbonate, raising the local pH and triggering carbonate precipitation [9, 10]. To reduce costs and expand the applicability of MICP, it is essential to identify robust and highly active strains that can grow in inexpensive culture medium and remain effective in adverse pH and salt concentrations. High‐throughput screening methods that directly assess enzymatic activity at the single cell level offer a compelling approach to identify such robust ureolytic microorganisms.

Fluorescence‐activated droplet sorting (FADS) is a high‐throughput screening method that enables the microfluidic sorting of single cells encapsulated in picoliter droplets. Because extracellular enzymatic products are retained inside droplets [11], this technique allows for screening of molecule‐secreting cells that could not be isolated using conventional fluorescence‐activated cell sorting (FACS). FADS has been successfully applied to screen enzymes such as peroxidases [12], galactosidases [13], cellulases [14], and for microbial cellulose production [15], facilitating the discovery of novel enzymes and improved variants through directed evolution and environmental bioprospection. Compared to multiwell colorimetric tests or urease plates that are currently used to isolate ureolytic microbes, FADS offers the potential to greatly expand the repertoire of ureolytic strains by harnessing the vast functional diversity of microorganisms available in environmental samples [16, 17, 18]. To leverage this potential, a biochemical assay for the fast quantification of urease activity in droplets needs to be developed. Although surrogate substrates are commonly used as fluorogenic probes for the enzymatic activity, they often show low specificity, poor kinetics [19] and have not yet been developed for urease detection. Accordingly, pH‐based assays using native urea remain the most direct and label‐free approach to detect ureolytic activity.

pH‐based assays are often used to assess ureolytic activity in bulk or microplate formats and have also been applied to investigate the precipitation of inorganic particles within droplets [20, 21, 22]. In a recent study, purified urease encapsulated in double emulsions was shown to generate tunable pH pulses via controlled diffusion, enabling mineralization with temporal precision [22]. In another exemplary work, ureolytic bacteria were encapsulated in microfluidic droplets to study MICP processes under controlled conditions [20, 21]. Notably, enzymatic hydrolysis of urea has been shown to induce pH changes and calcium carbonate precipitation in cell‐free adjacent droplets [21]. These observations suggest that products of the hydrolysis reaction, such as ammonia, diffuse between droplets and interfere with signal localization, affecting the specificity of the pH assay [23, 24]. Such chemical crosstalk between droplets may explain why no high‐throughput method has yet been reported for detecting microbially induced pH changes in droplets at the single cell level. pH‐based assays in droplets have so far been limited to low‐throughput applications, such as sorting by interfacial tension (SIFT), which relies on pH‐sensitive surfactants to passively separate metabolically active cells based on changes in interfacial tension rather than fluorescence readouts [25]. These limitations underscore the need for a robust, pH‐based fluorescent assay that can reliably identify ureolytic activity at high throughput while accounting for diffusional crosstalk.

Herein, we present a high‐throughput microfluidic platform for the screening of microorganisms using the cell‐in‐bead‐in‐droplet (CiBiD) architecture for controlled microbial encapsulation. To demonstrate this technology, the CiBiD concept is used to selectively sort ureolytic bacteria based on local pH changes. In this illustrative case study, pH changes arising from diffusional crosstalk are minimized by engineering cell‐laden droplets with high ureolytic activity, and introducing a buffering system into all droplets to neutralize diffusing ammonia. The platform is demonstrated using genetically engineered *E. coli* as a model ureolytic bacterium and a fluorescent dye as a pH indicator. We first describe the two‐step process used to generate cell‐laden hydrogel beads that are later emulsified to form cell‐laden droplets with high ureolytic activity. Using confocal microscopy, we study bacterial proliferation inside the beads and examine how colony growth influences enzymatic activity. We then systematically evaluate how bacterial concentration and buffer composition affect local pH changes in both cell‐laden and empty microfluidic droplets. Finally, we demonstrate CiBiD's ability to enrich ureolytic strains from a mock microbial community and from an environmental sample, highlighting its potential as a high‐throughput platform for screening pH‐shifting microorganisms.

## 2 Results and Discussion

**FIGURE 1:**
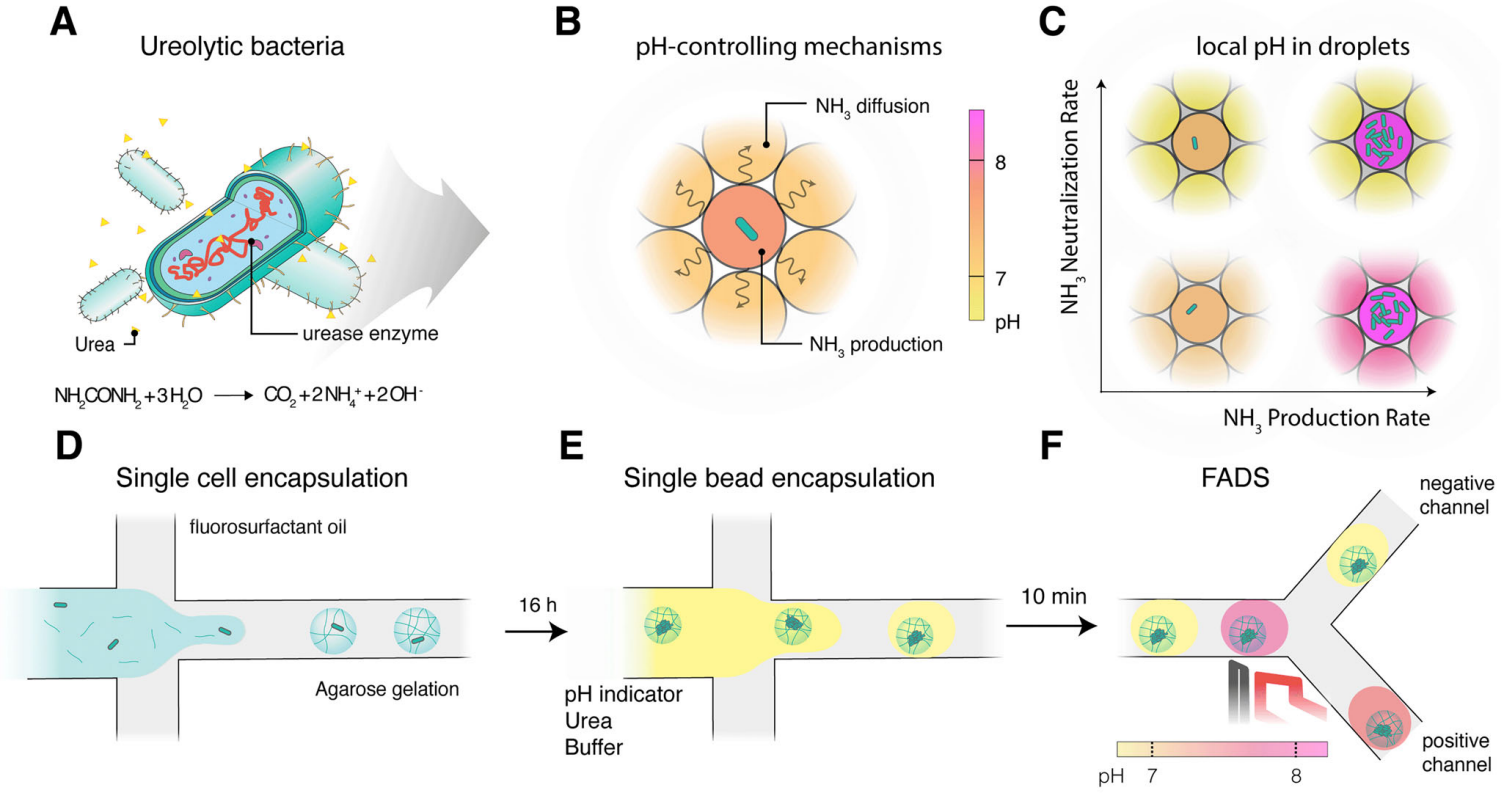
Schematic illustration of a pH sorting assay for high‐throughput screening of ureolytic bacteria. (A) Ureolytic bacteria express the enzyme urease, which catalyzes the hydrolysis of urea into carbon dioxide, ammonium, and hydroxide ions, leading to a local pH increase. This process is relevant for applications such as microbially induced calcium carbonate precipitation (MICP). (B) When encapsulated within single emulsion droplets, ureolytic bacteria can change the local pH of cell‐laden and neighboring droplets through the production, diffusion and neutralization of ammonia or ammonium ions. (C) Expected pH patterns in assemblies of active and neighboring droplets. The local pH environment is determined by the balance between ammonia production in the cell‐laden droplet and ammonia neutralization in adjacent droplets. High pH differences between cell‐laden and neighboring droplets are expected for high ammonia production and neutralization rates. (D,E) To generate CiBiDs, (D) single bacterial cells are first encapsulated in agarose beads using a microfluidic single emulsification process. After growing the bacteria to colonies, (E) the agarose beads are re‐encapsulated into single‐emulsion droplets containing urea, buffer, and a pH indicator to enable pH‐based detection of urease activity. (F) Fluorescence‐activated droplet sorting selectively isolates highly active ureolytic bacteria based on pH‐induced fluorescence changes. Strong ammonia production by highly active bacteria combined with high ammonia neutralization by the buffer should lead to a detectable pH shift.

Ureolytic bacteria are soil‐dwelling microorganisms capable of hydrolyzing urea into carbon dioxide, ammonium, and hydroxide ions (Figure 1A) [26]. This chemical reaction increases the pH of the aqueous phase to values close to 9 [27], causing the precipitation of calcium carbonate particles if calcium ions are present in the solution. Such bacteria‐induced precipitation has been exploited in several applications, such as self‐healing concrete, wastewater treatment, bioremediation and soil stabilization [3, 4, 5, 6, 7, 8]. To accelerate the precipitation process, microorganisms with high ureolytic activity are often desirable in these applications. Given the pH increase resulting from urea hydrolysis, screening for ureolytic microorganisms typically relies on detecting pH changes in the culture medium [17]. While many colorimetric and fluorescence assays can be used to detect pH changes, high‐throughput microfluidic screening of ureolytic bacteria is hindered by chemical crosstalk between droplets, which causes pH signal interference and prevents accurate sorting (Figure 1B). Crosstalk arises from the inter‐droplet diffusion of uncharged ammonia (NH_3_), which preferentially partitions into the fluorinated oil compared to its ionic form (NH_4_^+^) [28].

The local pH in cell‐laden droplets is determined by the interplay between the rate of ammonia production within urease‐active droplets and the rate of ammonia diffusion and neutralization in adjacent droplets. A reliable pH‐based screening approach requires high contrast in absorption or fluorescence between ureolytic droplets and their neighbors. Achieving this contrast necessitates both high local ammonia production in ureolytic droplets, combined with rapid ammonia neutralization in adjacent droplets to compensate for diffusion‐induced crosstalk (Figure 1C). Increasing the ammonia production is often challenging due to the inherently low ureolytic activity of the encapsulated microorganisms. This challenge becomes more critical in single‐cell screening methods, which rely on the ureolytic activity of a single encapsulated microorganism.

Single‐cell encapsulation is crucial to maintaining the genotype‐phenotype link and ensuring accurate selection of high‐performing variants in directed evolution or bioprospection campaigns. However, preliminary experiments indicated that the NH_3_ production rate from single encapsulated cells is not sufficient to generate a detectable local pH change, since ammonia diffuses into adjacent droplets before raising the pH. Therefore, we hypothesized that a higher local cell density is required to increase ammonia production to detectable levels.

To test this hypothesis, we developed a two‐step encapsulation strategy that enables high local ammonia production in droplets while keeping the single‐cell feature of microfluidic screening methods. The idea is to grow the initial single cell into microcolonies of high ureolytic activity before the high‐throughput screening step. Following this “grow‐first, assay‐afterward” concept, high local ammonia production is achieved while keeping the genotype–phenotype linkage through the hydrogel beads. For this, single bacteria are first encapsulated in agarose hydrogel beads to spatially entrap each cell while also allowing for colony growth in a controlled microenvironment (Figure 1D). The agarose beads remain suspended in the original fluorinated oil phase for cell proliferation, and are only later de‐emulsified and re‐encapsulated to create CiBiDs.

After proliferation, the colony‐laden beads are re‐encapsulated into aqueous droplets, together with urea, a buffer, and a fluorescent pH indicator (Figure 1E). The presence of multiple urease‐active cells within each bead is expected to display a rapid pH increase upon urea hydrolysis. Combined with such a fast local pH increase, the buffer present in the droplets can neutralize the ammonia that diffuses to adjacent droplets. Using such an approach, we expected that only droplets containing highly ureolytic beads will generate a strong and localized pH shift within the time window of the droplet sorting step.

Using this 2‐step emulsification platform, it should be possible to selectively screen and enrich pH‐changing microorganisms using a fluorescence‐activated droplet sorter. In this approach, droplets containing highly ureolytic microorganisms are directed into a positive sorting channel based on their fluorescence signal, which corresponds to a pH above a defined threshold, while non‐ureolytic and empty droplets are discarded (Figure 1F). To achieve effective sorting, it is crucial that the pH difference between ureolytic and non‐ureolytic droplets is both substantial and sustained during the sorting procedure.

**FIGURE 2:**
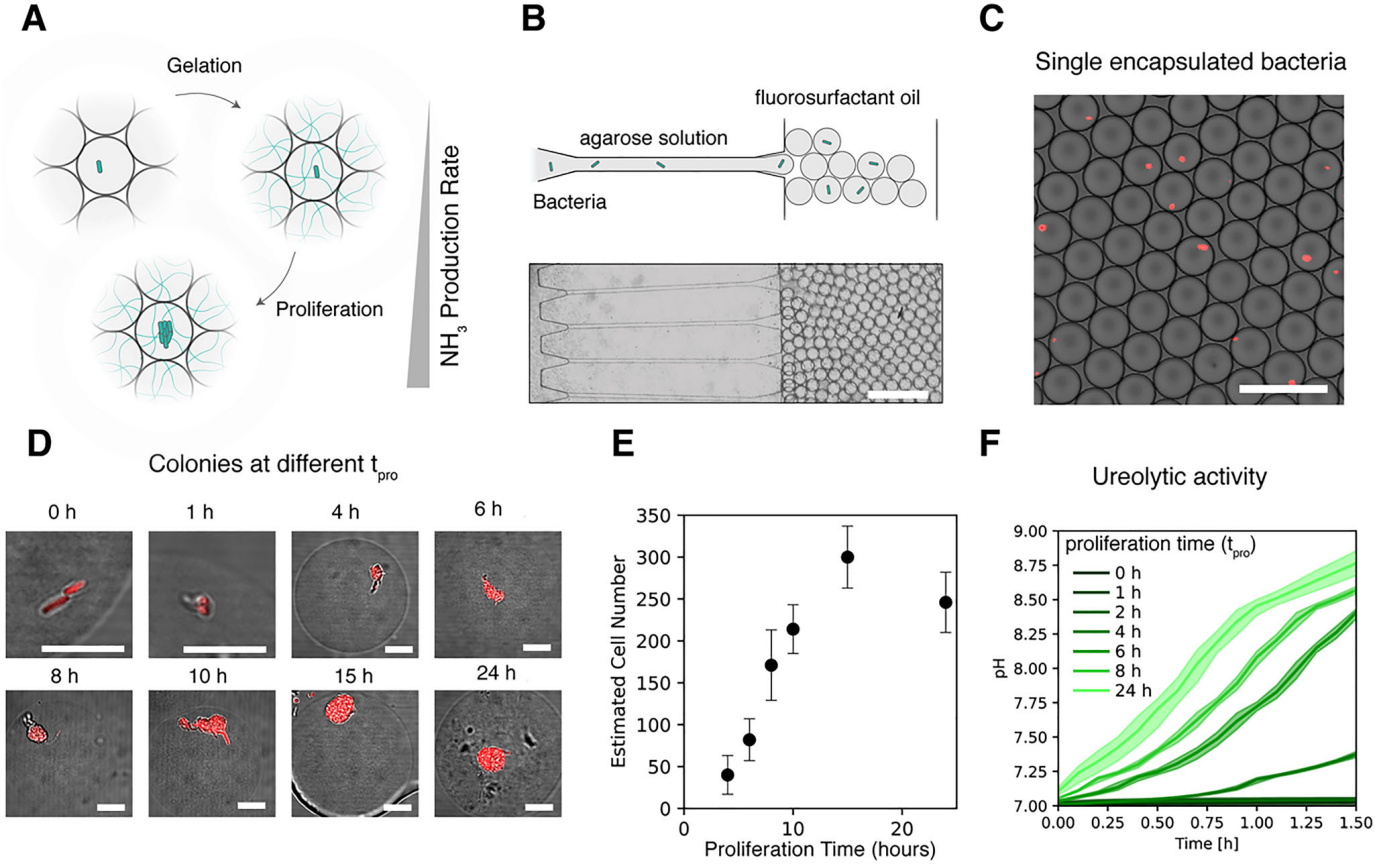
Encapsulation, growth, and ureolytic activity of *E. coli*‐laden hydrogel beads. (A) Schematic representation of single‐cell encapsulation in hydrogel beads, cell proliferation into microcolonies, and the expected increase in ammonia production rate. (B) Microfluidic step emulsification device used to generate monodisperse hydrogel beads containing *E. coli*. The top panel illustrates the droplet formation process, while the bottom image shows part of the actual parallelized step emulsifier. Scale bar corresponds to 200 µm. (C) Confocal microscopy image confirming single‐cell occupancy within hydrogel beads. *E. coli* expressing RFP are visualized as red dots. Scale bar corresponds to 100 µm. (D) Representative confocal images showing colony growth over different proliferation times (t_pro_) from 0 to 24 h. Bacteria proliferate within hydrogel beads, forming dense microcolonies. Scale bar corresponds to 10 µm. (E) Quantification of cell proliferation in beads in terms of estimated cell number as a function of proliferation time. Error bars represent standard deviations from 3 replicate measurements (n = 3). (F) pH increase over time, indicating urease activity of cell‐laden beads subjected to different proliferation times.

The feasibility of the proposed screening approach was evaluated by first studying the encapsulation and growth of single cells into microcolonies inside hydrogel beads. Because of their high water content and permeability to nutrients (see Supporting Information), hydrogel beads provide an ideal environment for the encapsulation and proliferation of single cells into dense microcolonies thereby enhancing ammonia production (Figure 2A). To illustrate this, we encapsulated engineered *E. coli* as a model ureolytic bacterium inside agarose beads. The bacterium was engineered to express urease and red fluorescent protein (RFP), allowing for both pH modulation and fluorescence‐based detection. Cell‐laden hydrogel beads were produced by suspending bacteria in a 1.5% liquid agarose solution and injecting the resulting suspensions into a microfluidic step emulsification device to generate monodisperse droplets. The obtained droplets were subsequently cooled to 4°C for 10 min to induce gelation of the agarose solution (Figure 2B). Beads were generated at a frequency of 2,000 Hz, enabling the production of millions of uniformly sized droplets within 10 min. The cell occupancy of hydrogel beads was assessed using confocal microscopy imaging, by placing a small fraction of the produced beads into a cell counting chamber. The results show bright RFP fluorescence in several of the imaged beads, confirming that the proposed protocol allows for the successful encapsulation of bacteria at the single cell level (Figure 2C).

Encapsulated bacteria proliferated into colonies of varying sizes and cell numbers, depending on the duration of the proliferation step. To assess the proliferation behavior of the entrapped bacteria colonies, hydrogel beads were incubated for different time periods and imaged using confocal microscopy. For imaging, the incubated hydrogel beads were transferred to water and placed between two glass slides (Figure 2D). The volume of each microcolony was quantified using image analysis, and the estimated cell count was determined by dividing the total colony volume by the average volume of a single bacterium. To quantify the growth of the entrapped microcolonies, we plotted the estimated cell counts against proliferation time (Figure 2E). The results revealed a rapid increase in bacteria number within the first 15 h of proliferation until a maximum of approximately 300 cells per bead is reached. After 24 h, the cell count declined to around 250 cells, which might result from bacteria leaving the hydrogel beads. This explanation is supported by optical microscopy images that show bacteria escaping the hydrogel when colonies reach the bead surface (Video S1).

The controlled growth of microcolonies inside agarose beads provides an effective method to tune ammonia production rate by the entrapped microorganism. To evaluate the effect of cell counts on ammonia production rates, we measured pH changes in a controlled urea‐rich environment using a colorimetric pH dye. In this experiment, ∼100,000 hydrogel beads were incubated in oil for different durations and afterward transferred to water before resuspension in aqueous medium containing 25 mM sodium phosphate buffer and 500 mM urea. The optical absorbance of the resulting mixture was analyzed in a plate reader to assess the rate of ammonia production by the grown colonies. Using a pre‐established calibration curve (Figure S1), the absorbance ratios at 435 nm and 560 nm were converted into pH values for beads previously subjected to different proliferation times.

The results showed that beads subjected to longer proliferation times exhibited a more rapid pH increase, indicating enhanced urease activity (Figure 2F). Data indicate that the cell‐laden beads need to be incubated for at least 8 h to enable a pH increase above 8 within the first 60 min of an experiment. Such a proliferation time leads to approximately 170 cells on average for each bead (Figure 2E). In contrast, beads containing fewer than 50 cells displayed no detectable pH rise within 1.5 h, indicating that a minimum cell density is required for measurable ammonia production. These findings confirm the hypothesis that high bacterial loads within droplets are essential for achieving local pH shifts.

**FIGURE 3:**
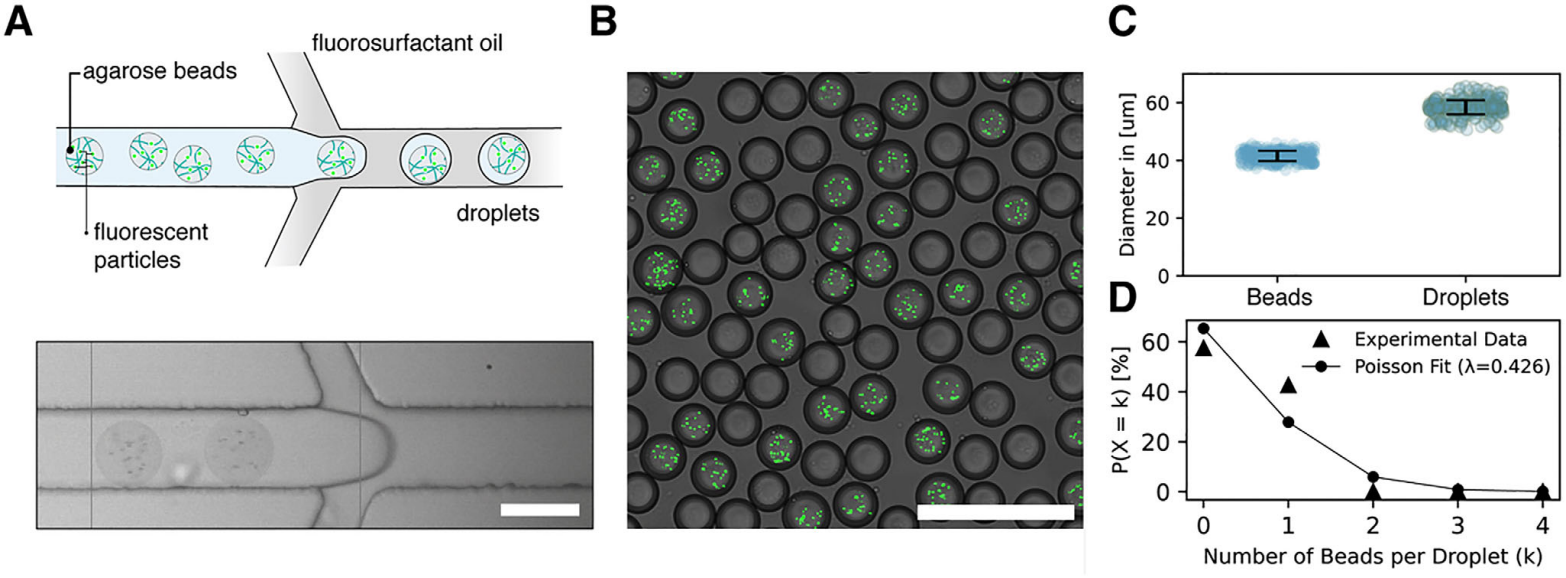
Encapsulation of particle‐laden beads in single emulsion droplets. (A) Schematic representation (top) and brightfield image (bottom) illustrating the re‐encapsulation process, where agarose beads loaded with fluorescent particles are emulsified into single‐emulsion droplets using a flow‐focusing microfluidic device. Scale bar corresponds to 50 µm. (B) Confocal microscopy image of the resulting droplets containing particle‐laden beads. Brightfield imaging is used to visualize the droplets, whereas fluorescence imaging enables quantification of bead occupancy. Scale bar corresponds to 200 µm. (C) Size distribution analysis of the agarose beads and the single‐emulsion droplets obtained from confocal images. Error bars represent standard deviation (n = 317 for droplets and n = 332 for beads). (D) Encapsulation efficiency analysis comparing experimental bead occupancy within droplets to the probability of occupancy given by Poisson statistics. The experimental bead occupancy is measured by the percentage of droplets (*P*) with a given number of droplets (*k*). The Poisson distribution was calculated for the bead concentration (λ) of 0.426 used in the experiments.

A prerequisite for detecting local pH changes using FADS is the re‐encapsulation of agarose beads into aqueous droplets to generate CiBiDs. This ensures that most of the reaction products of urea hydrolysis remain physically confined, thereby minimizing crosstalk between droplets. To keep the genotype‐phenotype link established during bead formation, each droplet should encapsulate a single agarose bead. Such a condition can be challenging because of the comparable sizes of the hydrogel beads and droplets. To determine the efficiency and limitations of this process, we studied the encapsulation of hydrogel beads within single‐emulsion droplets using fluorescent particles as cell mimics. For this, agarose beads were loaded with fluorescent particles to aid visualization and subsequently emulsified using a flow‐focusing microfluidic device (Figure 3A). Since the fluorescent particles were solely used to detect the presence of beads inside droplets, the variations in the concentration of fluorescent particles inside each bead had no impact on the output of the experiment. After emulsification, the resulting bead‐laden droplets were collected, and a subset of the collected sample analyzed via confocal microscopy. Brightfield imaging enabled precise identification and size analysis of the droplets, while fluorescence imaging allowed assessment of the number of beads per droplet (Figure 3B).

The fluorescently tagged agarose hydrogel beads and their corresponding droplets exhibited well‐defined and narrow size distributions, with beads and droplets averaging 40 µm and 58 µm in diameter, respectively (Figure 3C). This high uniformity highlights the precision of the microfluidic encapsulation process and contributes to the creation of a well‐defined microenvironment for the assessment of the biological activity of the encapsulated microcolonies. To quantify the encapsulation efficiency, we analyzed 1,127 droplets and compared the obtained distribution to Poisson statistics (Figure 3D). The analysis showed that 480 droplets contained a single agarose bead, while the remaining 647 droplets were empty. Notably, no droplets were found to contain more than one bead. The observed fraction of droplets with single‐bead occupancy is higher than predicted from Poisson statistics, suggesting that our method exceeds the statistical limit typically observed in random encapsulation processes. We hypothesize that this deviation arises from the size ratio between the agarose beads (∼40 µm) and the microfluidic channel (∼50 µm). The close confinement within the channel likely imposes spatial ordering, reducing stochastic loading variability and leading to enhanced single‐bead encapsulation efficiency [29]. The CiBiD platform's reliable loading of one cell‐laden bead per droplet makes it possible to detect the pH shift generated by each individual bacterial colony.

**FIGURE 4:**
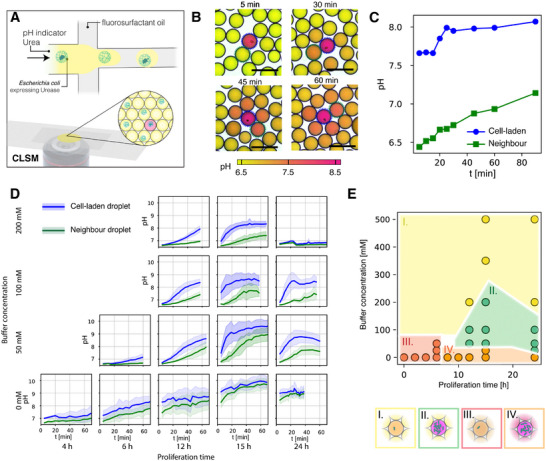
CiBiD microfluidic platform highlighting the conditions required for the detection of local pH changes induced by bacterial activity. (A) Schematic representation of the microfluidic workflow used to generate CiBiDs. Agarose beads loaded with *E. coli* colonies expressing urease are encapsulated in single emulsion droplets of water containing a pH indicator and urea. Droplets are transferred into a cell counting chamber, and their individual pH changes monitored over time using confocal laser scanning microscopy (CLSM). (B) Time‐lapse confocal images showing pH changes within droplets. Active droplets (circled blue) containing urease‐expressing bacteria reach high pH within the first 5 min, while neighboring droplets (circled green) exhibit an increase of pH over 60 min. Scale bars represent 100 µm. (C) Quantification of pH within cell‐laden and neighboring droplets over time. (D) Representative data displaying pH progression in cell‐laden and neighboring droplets under varying buffer concentrations (0–200 mM) and proliferation times (4–24 h). Blue and green lines indicate the average pH values for active droplets and their neighboring droplets with the highest pH, respectively. Shaded regions indicate standard deviations (*n* = 5 – 20 replicates per condition). (E) Map displaying the set of proliferation times and buffer concentrations needed to generate distinct local pH patterns within an assembly of active and neighboring droplets. The green region indicates the optimal conditions for pH change detection during sorting.

To identify the conditions allowing for the detection of local pH changes, we monitored the pH evolution of urea‐containing droplets with and without bacteria‐laden beads over time. pH detection was possible by adding fluorescent or colorimetric dyes in the aqueous phase prior to emulsification (Figure 4A). After encapsulation, droplets were collected on a cell‐counting chamber to form a monolayer for time‐lapse imaging in an optical microscope. The results show that bacteria‐laden droplets display a pH of approximately 7.5 after the first 5 min of the experiment, which is significantly higher than the pH of 6.5 detected in the cell‐free adjacent droplets (Figure 4B). This high initial pH difference reduces over the first 60 min due to the diffusion of NH_3_ from the bacteria‐laden droplet to the cell‐free neighbors. Notably, droplets positioned farther from the bacteria‐containing droplet took longer to change color, consistent with diffusion‐limited crosstalk.

Using pre‐defined calibration curves, we tracked the difference in pH between cell‐laden and a cell‐free adjacent droplet as a function of time (Figure 4C). The pH difference decreases from 1.2 to 0.8 in the first 90 min of the experiment. These findings indicate that it is possible to detect local pH changes in bacteria‐laden droplets. Moreover, the colorimetric change associated with this pH change should be sufficiently large to enable effective sorting of droplets containing urease‐active bacteria.

The ideal time window for pH‐based droplet sorting depends on the proliferation time used for bacterial growth within the beads, and the buffer concentration in the aqueous phase. Longer proliferation times increase bacterial density, enhancing the rate of NH_3_ production and thereby accelerating pH changes. Conversely, higher buffer concentrations are expected to increase the neutralization rate of NH_3_ in the droplets, thereby counterbalancing the pH change induced by the bacteria. To investigate this interplay, we tracked local pH changes in multiple droplets using a fluorescent dye and a confocal microscope. The proliferation time was varied between 0 and 24 h, while the buffer concentration ranged from 0 to 500 mM. To obtain statistically significant data, we measured the pH of at least five cell‐containing droplets along with their neighboring droplets over time (Figure 4D). Results show that longer proliferation times increase the absolute pH of the cell‐laden droplets, while keeping the pH gap between cell‐laden and adjacent droplets constant. This is attributed to the increased bacterial load and hence higher ureolytic activity at longer proliferation times. In contrast, increasing buffer concentration reduced the absolute pH but increased the pH gap, indicating effective mitigation of NH_3_ crosstalk in neighboring droplets.

For high‐throughput droplet sorting based on local pH changes, two critical conditions must be fulfilled. First, the pH increase within the cell‐laden droplet must be sufficiently strong to indicate high bacterial activity. We arbitrarily define this condition as fulfilled if the pH of the cell‐laden droplet exceeds 8 within the first 30 min. Second, the pH difference between cell‐laden and neighboring empty droplets must be both detectable and sustained over the time interval typically required for droplet sorting. This condition is met if the pH difference between cell‐laden and adjacent droplets remains statistically significant for at least 30 min within the first hour of incubation. We consider the pH difference to be statistically significant if there is no overlap between the standard deviations of the measured values. This analysis delineates distinct scenarios for the local pH dynamics inside droplets, allowing us to experimentally identify the optimal parameter space where both conditions are satisfied (Figure 4E).

Experiments revealed that proliferation times between 10 and 15 h and buffer concentrations between 50 and 200 mM lead to optimum conditions for pH‐based droplet sorting (Figure 4E, green region). While the need of a minimum proliferation time and buffer concentration confirms our initial hypothesis, the upper limits identified in the experiments indicate additional effects controlling the absolute pH of the active droplet and the pH gap between droplets. Indeed, data show that buffer concentrations above 200 mM lead to an undesirable drop in the absolute pH of the cell‐laden droplet, probably due to fast ammonia neutralization. Likewise, an proliferation time longer than 15 h also reduces the absolute pH of the cell‐laden droplet at the high buffer concentration of 200 mM. This effect likely results from bacterial escaping from the beads due to growth‐induced local rupture of the agarose hydrogel (Supplementary Video S1). By adjusting the buffer concentration and the proliferation time at intermediate ranges, we identified key parameters that enhance NH_3_ production in cell‐laden droplets while simultaneously increasing NH_3_ neutralization in neighboring droplets. This provides a suitable parameter space for screening urease‐active microorganisms via pH‐based droplet sorting.

**FIGURE 5:**
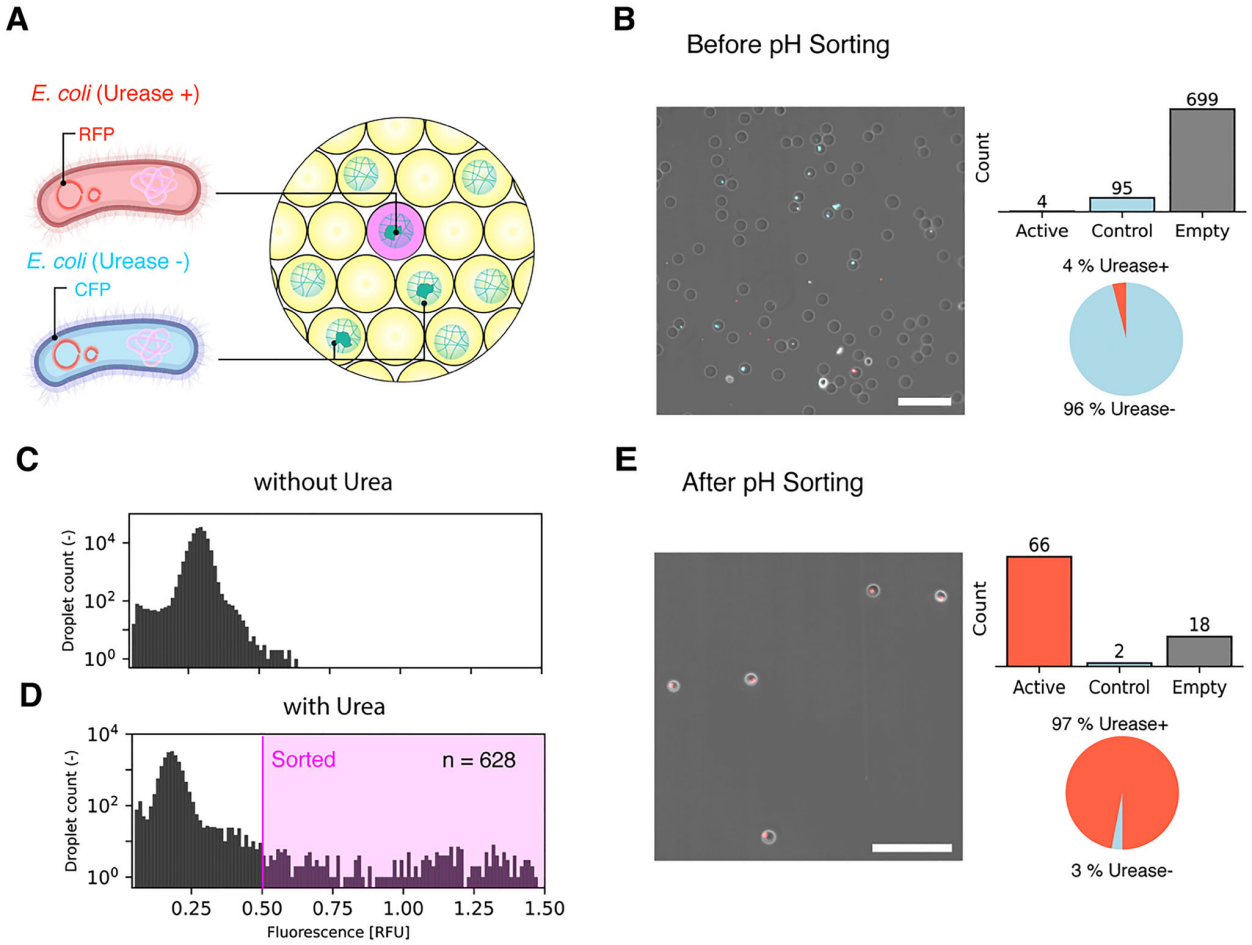
Enrichment of ureolytic bacteria via local pH increase using CiBiDs in a fluorescence‐activated droplet sorting device. (A) Schematic representation of the encapsulated mock consortium (CiBiDs), consisting of two genetically distinct *E. coli* strains: a urease‐expressing active strain labeled with red fluorescent protein (RFP, Urease^+^) and a control strain expressing cyan fluorescent protein without urease (CFP, Urease^−^). (B) Confocal microscopy image of the agarose beads before the second encapsulation step, showing RFP‐positive (Urease^+^) and CFP‐positive (Urease^−^) bacterial colonies within agarose beads. The accompanying bar graph and pie chart illustrate the initial consortium composition, with 4% urease‐positive active colonies and 96% control colonies in the occupied droplets before sorting. Scale bars correspond to 200 µm. (C) Histogram displaying the fluorescence of CiBiDs prepared in the absence of urea. Fluorescence was measured via a photomultiplier tube (PMT) after 488 nm laser excitation during droplet flow in the FADS setup. (D) Fluorescence histogram of CiBiDs in the presence of urea, showing a clear right‐shifted subpopulation of droplets featuring higher fluorescence induced by local pH increase. The magenta region indicates droplets above the threshold selected for sorting (*n* = 628). (E) Post‐sorting microscopy image, confirming the successful enrichment of urease‐expressing *E. coli*. The corresponding bar graph and pie chart reveal a substantial increase in the fraction of RFP‐positive (Urease^+^) active colonies, reaching 97% of the sorted population. Scale bars correspond to 200 µm.

To demonstrate the suitability of the pH‐based assay for the high‐throughput screening of ureolytic bacteria, we tested its sorting efficiency using a FADS device. The droplet sorting process was tested by designing a mock microbial consortium consisting of two genetically distinct *E. coli* strains: a urease‐expressing strain labeled with red fluorescent protein and a non‐ureolytic control strain expressing cyan fluorescent protein (CFP) (Figure 5A). Each strain was separately encapsulated in agarose beads and subsequently emulsified, followed by incubation at 37°C for 15 h. After incubation, the resulting droplets were de‐emulsified and the agarose beads were combined to establish a consortium with a final 1:20 ratio of urease‐positive to urease‐negative cells. The fraction of ureolytic bacteria in the consortium (5%) falls within the range 1.4%–17% of ureolytic microrganisms previously estimated for distinct soil samples using amplicon sequencing data [30]. To evaluate whether the RFP and CFP signals can be used to assess the composition of the mock consortium, we first imaged the encapsulated colonies in a confocal microscope. Among 798 total beads analyzed, 4 contained RFP‐positive (urease‐expressing) colonies, 95 contained CFP‐positive (non‐ureolytic) colonies, and 699 were cell‐free. These data confirm that 96% of the cell‐laden beads were urease‐negative, while 4% contained urease‐positive cells, in close agreement with the 1:20 ratio expected from the initial mock composition (Figure 5B).

We then evaluated the assay's ability to selectively enrich urease‐active bacteria using FADS. The 1:20 consortium was re‐encapsulated in an aqueous phase containing 0.5 m urea, 50 mM sodium phosphate buffer, and 0.5 mM HPTS, a pH‐sensitive fluorescent dye, to generate CiBiDs. As a control, droplets were also prepared with buffer and dye but without urea. We note that the sorting process is based on the fluorescence of the pH‐sensitive HPTS dye, whereas the fluorescence signals generated by the recombinant proteins CFP and RFP are solely used to quantify the ratio of urease‐positive and urease‐negative cells before and after the sorting process. The encapsulated microorganisms were incubated for 15 h to reach the conditions required for high and sustained local pH increase (Figure 4E). After a 15‐min activation period, a fraction of the encapsulated mock consortia was processed through the FADS system. We note that the activation step is long enough for the ureolytic reaction to take place, while sufficiently short to ensure oxygen is not depleted from the urease‐producing cells [31]. In the FADS setup, droplets are interrogated by a laser, and the resulting fluorescence is detected by a photomultiplier tube (PMT), which converts the emission signal into a voltage peak used for sorting decisions. The PMT voltage signal is used to quantify the pH‐dependent fluorescence of the HPTS dye within the droplet. As expected, droplets lacking urea showed minimal HPTS fluorescence due to the absence of pH change (Figure 5C). In contrast, droplets containing ureolytic bacteria and urea exhibited elevated HPTS fluorescence levels, clearly distinguishable from inactive or empty droplets. Based on a user‐defined fluorescence threshold, the urease‐positive droplets are actively sorted into a positive collection outlet using dielectrophoresis, while non‐fluorescent droplets are discarded (Figure 5D).

To evaluate the sorting efficiency of the platform, approximately 360,000 droplets were processed through the sorter over 30 min. From this large initial pool, 628 droplets surpassed the user‐defined HPTS fluorescence threshold (0.5 RFU) and were directed into the collection outlet (Figure 5D). The sorted single emulsion droplets were then de‐emulsified, and a fraction of the sample imaged using a confocal scanning microscope and analyzed for the presence of RFP‐ or CFP‐labeled colonies (Figure 5E). Out of 86 sorted beads, 66 contained RFP‐labeled (urease‐positive) bacteria, 2 contained CFP‐labeled (urease‐negative) bacteria, and 18 were cell‐free. This resulted in a substantial enrichment of ureolytic cells, with 97% of the sorted cell‐laden beads containing RFP‐positive colonies (Figure 5E). This corresponds to an overall purity of 76% across all sorted beads. The observed 25‐fold enrichment underscores the efficiency of the proposed pH‐based droplet sorting method in selectively isolating ureolytic bacteria from mixed microbial populations.

Our enrichment levels are comparable to those achieved using fluorescence‐based screening assays that do not involve local pH changes. For example, fluorescence‐based screening of amylase‐containing droplets [32] resulted in ∼75% purity at a similar active‐to‐inactive dilution ratio, but with a slightly lower overall enrichment factor of approximately 20. Similarly, microfluidic sorting of cellulase‐producing cells using FADS showed that the purity and enrichment factor depend strongly on the initial active‐to‐inactive dilution ratio [33]. For a comparable dilution ratio of 1:20 active‐to‐inactive cells, we estimate a purity of 79% and an enrichment factor of 16 after sorting of the cellulase‐producing cells. This comparative analysis reveals that the CiBiD platform is on par with state‐of‐the‐art enrichment assays, demonstrating the effectiveness of the proposed pH‐based assay for the high‐throughput selection of pH‐changing microorganisms. Other microorganisms that can potentially be screened using this platform include proteolytic, sulfur‐oxidizing, lactic acid, and acetic acid bacteria.

**FIGURE 6:**
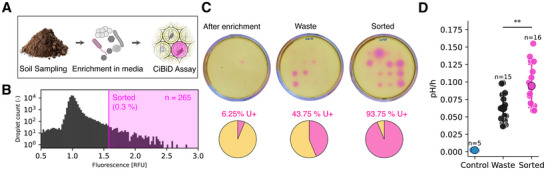
High‐throughput bioprospection of ureolytic bacteria from the soil. (A) Schematic representation of the workflow for the isolation of ureolytic microorganisms from the soil. After soil sampling, ureolytic bacteria were enriched in media and afterward isolated using the CiBiD assay. (B) Fluorescence histogram of soil CiBiDs in the presence of urea, showing a clear right‐shifted subpopulation of droplets featuring higher fluorescence induced by local pH increase. The magenta region indicates droplets above the threshold selected for sorting (*n* = 265). Sorted beads were recovered on urea‐peptone agar and incubated at 30°C to allow the formation of single colonies (C) Images of urea agar plates after 8 h of incubation at 30°C, showing ureolytic activity of isolates recovered from the enriched, wasted, and sorted fractions. For each condition, sixteen colonies were grown in liquid urea‐peptone medium to an OD_600_ > 0.5, and 5 µL of each culture was spotted onto urea–peptone agar. Ureolytic colonies produced pink halos due to local pH increase. The isolates differed markedly in the proportion of active colonies: after enrichment = 1/16, waste = 7/16, sorted = 15/16. (D) Quantification of bulk urease activity of isolates from the sorted, waste, and control fractions. The control isolates were obtained by direct plating of soil dilutions on urea–peptone agar without prior encapsulation or sorting. pH changes were monitored in 96‐well plates containing 0.5 m urea and 0.1 m HEPES using phenol red as a pH indicator. As expected, the negative control isolates displayed negligible pH changes in bulk. Waste isolates exhibited low but measurable urease activity, while sorted isolates showed significantly higher urease activity (unpaired two‐tailed t‐test, p = 0.0036), confirming that the CiBiD assay effectively enriched for highly ureolytic bacteria.

Beyond engineered bacteria, the CiBiD platform can also be applied for screening and bioprospection of ureolytic microorganisms from environmental samples (Figure 6A). To illustrate this, a soil sample collected from a forest near Hönggerberg (Zürich, Switzerland) was enriched for ureolytic bacteria using the CiBiD technology. The collected soil was first immersed in sodium acetate–glucose–peptone–urea medium and incubated at 30°C for 16 h to enrich the cell population with ureolytic microorganisms. After sedimentation and filtration through a 5 µm mesh, the microbial suspension was encapsulated in 1.5 wt % agarose beads and incubated for 24 h to form microcolonies. The beads were subsequently re‐encapsulated in single emulsion droplets containing 0.5 m urea, 40 mM sodium phosphate buffer, and 0.5 mM HPTS for pH‐based fluorescence detection. Approximately 80,000 droplets (3,000 microcolonies) were analyzed by FADS within 15 min. The most fluorescent droplets were collected based on a threshold fluorescence intensity (Figure 6B). The threshold was chosen such that 0.3% of droplets (10% of microcolonies, n = 265) could be sorted. Such droplets featured the most alkaline microenvironments and thus carried microorganisms with the strongest urease activity.

After sorting, the droplets were de‐emulsified and plated on a urea–peptone agar substrate to recover single colonies. To evaluate the efficiency of the microfluidic bioprospection process, three sets of colonies were isolated from samples that were: (1) initially enriched but not sorted, (2) enriched and disposed (waste), and (3) enriched and selected (sorted). Sixteen isolates from each condition (after enrichment, waste, and sorted) were grown to optical density, OD_600_, higher than 0.5 and spotted (5 µL) onto urea‐laden agar plates. The agar plates also contained the pH indicator phenol red, which shifts from yellow to pink when the pH changes from neutral to alkaline. The ureolytic activity of the colonies was assessed after 8 h of incubation at 30°C. The experiments revealed that ureolytic colonies appeared as pink halos due to local pH increase (Figure 6C). Notably, the proportion of ureolytic colonies increased from 6.25% after enrichment to 93.75% after sorting, indicating that the microfluidic bioprospection process led to strong enrichment of urease‐active bacteria.

Interestingly, a considerable fraction of the waste isolates (43.75%) also displayed ureolytic activity, suggesting that our initial enrichment step had already favored the proliferation of urease‐positive species in the microbial population. The pre‐selection of such ureolytic bacteria was likely favored by the use of urea‐containing medium during the initial enrichment phase and by the high nutrient content available during microcolony formation in agarose beads. Despite this pre‐selection effect, bulk urease assays performed in 96‐well plates revealed that the sorted isolates exhibited significantly higher pH increase rates than waste isolates (p = 0.0036, unpaired t‐test; Figure 6D). The enhanced activity of the sorted isolates is even clearer when compared with a control group. The latter consisted of single colonies obtained by directly plating serial dilutions of the soil suspension onto urea–peptone agar plates, without prior encapsulation or sorting.

This shows that the CiBiD platform not only identifies ureolytic bacteria from complex environmental samples but also efficiently isolates those with the highest urease activity. Taken together, this comparative analysis demonstrates that the CiBiD platform performs on par with state‐of‐the‐art enrichment workflows, while benefitting from the single‐cell screening capabilities offered by droplet microfluidic platforms. This validates the effectiveness of the CiBiD technology for the high‐throughput targeted selection of pH‐changing microorganisms from complex communities.

Compared to existing high‐throughput microfluidic screening methods [15, 34, 35, 36, 37, 38, 39, 40, 41, 42, 43], CiBiD provides a complementary strategy that balances phenotypic isolation, experimental simplicity, and downstream accessibility. Conventional single‐emulsion droplet sorting (FADS) enables very high throughput but is prone to crosstalk of diffusible metabolites and typically requires droplet rupture after sorting, which demands careful handling to maintain genotype–phenotype linkage. Double‐emulsion droplets compatible with FACS reduce molecular exchange but require more complex fabrication and operate at lower throughputs. Hydrogel bead–based approaches facilitate containment and downstream handling but typically lack a dedicated compartment for assay separation. Core–shell or gel‐shell droplet architectures further reduce molecular leakage but require increased material complexity and more demanding fabrication steps. By separating microbial growth from phenotypic readout while retaining the hydrogel bead as the physical unit of selection, CiBiD circumvents strong crosstalk effects while remaining experimentally accessible.

A central advantage of CiBiD is the preservation of a stable genotype–phenotype linkage throughout growth, sorting, and recovery. Because cells remain confined within monoclonal, cell‐laden beads, each bead can be treated as an individual, clonal entity after sorting. This feature enables extended workflows beyond single‐pass screening, including (i) iterative enrichment through repeated sorting rounds under identical selection pressure, (ii) sequential phenotyping using different assay chemistries, (iii) direct FACS‐based bead deposition into microtiter plates for downstream culturing or validation, and (iv) genomic or metagenomic analysis supported by the high DNA‐yield of bead‐confined microcolonies. In this sense, CiBiD functions as a modular screening platform rather than a one‐step sorting assay.

In terms of limitations, CiBiD introduces practical constraints that define its optimal use cases. Growth in agarose beads can be slower than in liquid culture, and colonies near the bead surface may locally disrupt the hydrogel matrix, allowing cell escape or growth at the oil–water interface. Such escape events can compromise genotype–phenotype linkage and lead to contamination in later assay stages. Encapsulation above the gelling temperature of ultra‐low‐melting agarose may limit compatibility with temperature‐sensitive organisms, and the second emulsification step requires careful control of bead size to maintain droplet stability and avoid clogging. Furthermore, maintaining single‐cell resolution necessitates low bead occupancy, resulting in a reduced fraction of assay‐relevant droplets.

Several mitigation strategies can be explored to circumvent these limitations. For example, cell escape events can be minimized by increasing agarose concentration and shortening incubation times to limit overgrowth. In addition, the number of free‐floating cells can be reduced by repeated washing and centrifugation at low centrifugal force (≈200 × g), which sediments agarose beads while leaving single bacterial cells in suspension [44]. Optimizing bead materials and preparation protocols in future research should enable further development of the method and help delineate the boundaries within which CiBiD is most effective [49, 50, 51, 52, 53, 54, 55].

Overall, CiBiD complements existing droplet‐ and bead‐based screening technologies by offering a robust compromise between throughput, phenotypic containment, and post‐sorting manipulability, making it particularly suited for multi‐stage microbial screening and discovery workflows.

## 3 Conclusions

We have developed a cell‐in‐bead‐in‐droplet (CiBiD) microfluidic platform that enables high‐throughput detection and sorting of ureolytic microorganisms based on localized pH changes. By accelerating both the production and removal of pH‐changing reaction products within cell‐laden droplets and their neighbors, the platform minimizes diffusional crosstalk and thereby allows for effective sorting of high‐performing microorganisms. To boost pH signal generation, bacteria are first encapsulated in agarose beads and proliferated to allow clonal expansion into microcolonies prior to substrate addition. This increases the local concentration of urease‐producing cells, thereby accelerating the local production of ammonia via urea hydrolysis. Crosstalk is suppressed by incorporating appropriate buffer concentrations in the droplets, which partially neutralize diffusing ammonia and stabilize the pH landscape across the droplet population. Optimization of these parameters led to strong pH shifts exclusively within active, cell‐laden droplets, thus ensuring accurate detection of microorganisms displaying high enzymatic activity. We quantified the efficacy of this pH‐based sorting approach by enriching active ureolytic bacteria from a mock consortium using high‐throughput fluorescence‐activated droplet sorting. By enabling high‐throughput sorting based on pH changes, this method provides a robust foundation for discovering and evolving ureolytic microorganisms for a wide range of biotechnological applications. We demonstrated this potential by successfully isolating highly ureolytic bacteria from environmental soil samples. Beyond its application to ureolytic microbes, the CiBiD platform introduces a broadly applicable microfluidic strategy for the high‐throughput detection of localized chemical signals in processes involving strongly diffusing molecules. Its design decouples microbial growth from assay chemistry, enabling clonal signal amplification, compatibility with minimal or chemically defined media, and accommodates challenging buffer conditions that would destabilize conventional droplet systems. Moreover, the use of hydrogel beads further simplifies downstream recovery of microorganisms for re‐analysis or cultivation. While this study focused on pH‐based sorting, the same architecture will be applicable to assays involving other extracellular products, providing a new tool to study dynamic cell interactions in complex microbial communities.

## 4 Experimental Section

### 4.1 Materials and Methods

#### 4.1.1 Materials

Agarose (Agarose Low Melting, Lot AS489940) was purchased from Apollo Scientific. Hydrofluoroether (HFE 7500) oil and 008‐FluoroSurfactant were ordered from RAN biotech. Unless otherwise stated, all other chemicals and reagents were purchased from Sigma‐Aldrich.

#### 4.1.2 Engineered Bacteria

A genetically modified *Escherichia coli* DH5α strain expressing both urease and red fluorescent protein (RFP) was used to study bacterial growth and urea hydrolysis within hydrogel beads. Urease expression was induced by adding isopropyl‐β‐D‐1‐thiogalactopyranoside (IPTG), which binds to the lac repressor, displacing it from the lac operator and thereby enabling transcription of genes under lac control. As a non‐ureolytic control, one strain was engineered to express cyan fluorescent protein (CFP). Details on the plasmid construction and urease expression system used in the *E. coli* vector are provided in the Supplementary Information.

#### 4.1.3 Cell Culturing

Engineered *E. coli* strains were employed in this study as urease‐expressing bacteria and as non‐ureolytic control microorganisms. Cells were cultured in lysogeny broth (LB) medium containing 5 g/L yeast extract, 10 g/L tryptone, 10 g/L NaCl, and 50 µg/mL kanamycin sulfate. 15 g/L agarose was added to the medium when needed for the formation of hydrogel beads. To induce urease expression, the medium was supplemented with 50 µM NiCl_2_ and 100 µM isopropyl‐β‐D‐1‐thiogalactopyranoside (IPTG). Working stocks were maintained on solid medium at 4 °C and renewed every four weeks from a −80°C frozen seed stock. To grow cells from the stocks, a single colony was inoculated into 0.5 mL of fresh medium in a 1.5 mL Eppendorf tube and incubated overnight at 38 °C until an optical density at 600 nm (OD_600_) of 0.8–1.0 was reached. OD_600_ was measured using a microplate reader (96‐well plates, 200 uL) with uninoculated medium as blank.

### 4.2 Microfluidic Experiments

#### 4.2.1 Generation of Hydrogel Beads

Low‐melting agarose (Apollo Scientific) was prepared at a concentration of 1.5 wt.% by adding it to deionized water (Milli‐Q) or growth medium and dissolving it using a microwave. Deionized water was used in the experiments performed to set up the platform (Figure 3), whereas growth medium was utilized for experiments with engineered *E. coli* (Figures 2, 4 and 5). After dissolution of the agarose, the solution was cooled to 40°C in a water bath. A 2 mL aliquot of the agarose solution was transferred to a 15 mL centrifuge tube and loaded with fluorescent particles or bacterial cells. To generate beads loaded with particles or cells, the aqueous suspension was emulsified in an adapted version of a previously reported microfluidic device [15, 45] using the fluorinated oil as outer phase. Microfluidic chip designs are available at https://github.com/ETH‐Complex‐Materials/CiBiD‐screening. For emulsification, the aqueous suspension was connected to an OB1‐Mk II pressure controller (Elveflow) and maintained in a water bath heated to 38°C. Next, an oil consisting of 2 wt.% FluoroSurfactant in HFE‐7500 was loaded into a 3 mL syringe and connected to a syringe pump (Harvard). Both phases were introduced into a parallelized step emulsification device, based on previously reported designs [15, 45, 46]. The device was operated at a flow rate of 500 µL/h for 10 min to form over one million droplets in a single run. The droplets were collected in 1.5 mL microcentrifuge tubes and cooled to 4°C for 15 min to solidify the agarose. During subsequent incubation at 38°C, the tubes were stored horizontally to promote uniform oxygen exposure across the emulsion. Detailed information about the operational parameters used for microfluidic bead generation are shown in Table S1.

### 4.3 Emulsion Breaking and Filtering

Prior to initiating the second emulsification step, the agarose hydrogel beads were further processed to remove the few larger beads that might form during the first emulsification step. This required transferring the beads from the oil phase into an aqueous phase. For this purpose, 500 µL of deionized water (Milli‐Q) and 30 µL of 1H,1H,2H,2H‐perfluorooctanol (Apollo Scientific) were added to the emulsion. The mixture was briefly vortexed for 5 s and then centrifuged for 1 min at 2550 RCF. The upper layer, containing the hydrogel beads suspended in water, was carefully collected with a pipette and passed through a 50 µm filter (pluriStrainer, Pluriselect) to remove large agarose beads.

### 4.4 Encapsulation of Agarose Beads Into Single Emulsion Droplets

CiBiDs were generated by encapsulating the monodisperse agarose beads in single emulsion droplets (Figure 3 and 4). For this, the hydrogel bead concentration was determined by counting beads in defined volumes using an optical microscope and, based on this information, the suspension was diluted to a final concentration of 4,000 beads/µL. Next, the suspension of beads was loaded into a 1 mL syringe and injected into a flow‐focusing microfluidic chip with channel width of 60 µm and a height of 50 µm to encapsulate the beads into single emulsion droplets. Microfluidic chip designs are available at https://github.com/ETH‐Complex‐Materials/CiBiD‐screening. 2 wt.% fluorosurfactant in HFE‐7500 was used as the continuous phase. Flow rates were set to 0.75 mL/h for the dispersed phase and 3 mL/h for the continuous phase. Droplets were collected for 10 min and stored in small 1.5 mL Eppendorf tubes with a closed top. For the short incubation time before the sorting step, the centrifuge tube was stored vertically at room temperature. Detailed information about the operational parameters used for microfluidic droplet generation are shown in Table S1.

### 4.5 Sorting of Beads Encapsulated in Single Emulsion Droplets

Droplet sorting was performed in a customized microfluidic device operated using LabVIEW code available from the Abate lab (https://github.com/AbateLab/sorter‐code). For the microfluidic sorting experiments (Figure 5 and 6), droplets were reinjected into the sorting device [47] using a pressure‐controlled system set to 120 mbar (LineUP Flow EZ, Fluigent). The droplets were transferred from the vial to the inlet of the sorting device using polyethylene tubing with inner (ID) and outer (OD) diameters of 0.86 and 1.32 mm, respectively (Scientific Commodities). Oil (HFE‐7500, 3 m) containing 1 wt % fluorosurfactant was delivered at a flow rate of 8 µL/min on both sides of the microfluidic channel to increase the spacing between the flowing droplets. While the long‐term operation of the sorting process was not investigated in this study, the device works reliably for the full 20–40 min sorting run. This is sufficient for sorting a sample at the high throughput of 200 droplets per second. Up to 6 samples with a total of 2,000,000 droplets can be processed through our device in a single day. Within the 5–10 sorting rounds performed in this work, clogging of the device was never a problem. Detailed information about the operational parameters used for microfluidic droplet sorting are shown in Table S1.

The incoming droplets were excited using a 488 nm solid‐state laser beam (Omicron Laserage Laserprodukte). The beam was expanded, shaped into a line, and focused on the chip using a 20×/0.45 NA Nikon S Plan Fluor ELWD objective. The fluorescence emission of the droplets was first filtered through a 30 µm pinhole and a 520/35 optical filter (Chroma Technology) and was finally captured using a photomultiplier tube (PMT, H10722‐20; Hamamatsu Photonics). In contrast to the confocal experiments (Figure 4), the sorting campaign was designed for high throughput rather than accuracy. This was achieved by using a single laser and an imaging timescale in the range of 1–10 milliseconds. Despite the lower accuracy during sorting, the high pH‐sensitivity of the HPTS dye was found to provide sufficiently strong fluorescence changes to effectively quantify the level of ureolytic activity of the encapsulated cells. The voltage signal from the PMT was sampled at a rate of 100’000 samples per second by a Field Programmable Gate Array (FPGA, NI PXI‐7842R; National Instruments) running a custom LabVIEW code. The code registered the signal from incoming droplets and, based on a user‐defined threshold, issued a series of tunable pulses that were fed to the chip via a high‐voltage amplifier (Trek 632B, Advanced Energy) for droplet sorting. During sorting, 25 to 35 pulses at a final voltage of 900 V were applied at a pulse frequency of 40 kHz, which corresponds to a train length of 0.6 to 0.8 ms.

### 4.6 Droplet Visualization

Confocal microscopy was utilized to analyse cell growth in beads (Figure 2), bead occupancy in droplets (Figure 3), local pH changes in droplets (Figure 4) and cell enrichment in mock consortia (Figure 5). To quantify these processes, microfluidically‐produced droplets were typically loaded into a hemocytometer (Countess cell counting chamber, Thermo Fisher) and visualized using a confocal laser scanning microscope (Leica TCS SP8). Fluorescent particles and proteins were excited using specific laser wavelengths, and their emissions were measured using HyD detectors. Fluorescent particles were excited with a 488 nm laser, with emission being detected between 515 and 525 nm. Red fluorescent protein was excited with a 552 nm laser, with emission being detected between 600 and 625 nm. Cyan fluorescent protein was excited using a 405 nm laser, with emission being detected between 415 and 475 nm. Additionally, a PMT transmission detector was used to capture gray images of the droplets. Droplet identification and diameter measurements were performed using MATLAB (R2023a, MathWorks) with the Circular Hough Transform algorithm. For fluorescence quantification, the average fluorescence intensity of each droplet was measured, and data exported as an Excel file for further analysis and visualization using Python. Droplets were classified as occupied by cells or particles if their fluorescence exceeded a manually defined threshold.

### 4.7 Quantitative pH‐Based Assays

#### 4.7.1 Bulk Urea Hydrolysis

To quantify urease activity at the population level, we monitored pH changes induced by the bulk hydrolysis of urea in the presence of *E. coli* engineered to express urease and RFP (Figure 2). Cells were encapsulated in agarose beads by injecting an agarose‐containing *E. coli* suspension with a mean number of cells per droplet of λ  =  0.2 into the step emulsification device. Culture medium was used as aqueous phase to prepare the cell suspension. Obtained droplets were collected for 1 min, yielding approximately 100,000 droplets per sample. After cooling‐induced gelation collected droplets were incubated at 37°C for varying durations (0, 1, 2, 4, 6, 8, and 24 h), and afterward de‐emulsified in 100 µL of deionized water (Milli‐Q). Each resulting solution was transferred to a 96‐well plate and mixed with urea (0.5 m), phenol red (0.05 mg/mL), and sodium phosphate buffer (0.625 mM, initial pH 6.5). Phenol red is convenient for the quantification of ureolytic activity in bulk solutions, since it provides visible readout for optical microscopy and plate reader measurements. Using the plate reader, absorbance at 600, 560, and 435 nm was measured every 2 min for 2 h, with intermittent shaking between readings. A pH calibration curve was generated using defined standards and fitted with a sigmoid function (Figure S1A,B) to translate absorbance changes into pH shifts.

### 4.8 Colony Growth of Encapsulated E. Coli

To assess bacterial proliferation within the agarose beads, colony growth was quantified at different proliferation times: 0, 2, 4, 6, 8, 12, and 24 h (Figure 2). For this, de‐emulsified beads were sandwiched between two glass slides and imaged using a confocal microscope (Leica TCS SP8) equipped with a 40x objective. Red fluorescent protein (RFP) signal was acquired in a z‐stack format with a step size of 1 µm to capture the full 3D volume of each colony. A custom image analysis pipeline (Figure S2A–C) was used to segment the colonies, calculate their total volume, and estimate cell counts. Cell counts were estimated by dividing the measured colony volume by the average volume of a single bacterium, as determined from individual cell measurements.

### 4.9 Local pH Change Measurement in Single Emulsion Droplets

Local pH changes in assemblies of droplets with (active) and without (neighboring) bacteria were quantified by monitoring the pH‐sensitive fluorescence of the co‐encapsulated dye (Figure 4). For this, urease‐expressing bacteria (RFP) were encapsulated in agarose beads at a loading density (λ) of approximately 0.2 and incubated at 37°C for varying durations (0, 1, 2, 4, 6, 8, 10, 12, 15, and 24 h). Droplet fusion or evaporation issues were not observed during incubation, which is performed off‐chip. After incubation, emulsions were broken, and the agarose beads were re‐encapsulated into single emulsions containing 0.5 M urea, 0.2 mM pyranine (8‐hydroxypyrene‐1,3,6‐trisulfonic acid, HPTS), and sodium phosphate buffer (initial pH 6.5) at varying concentrations. In this assay, HPTS was used as the ratiometric fluorescent pH‐sensitive dye. This dye is particularly suitable for the quantification of microbial activity inside droplets, since its pH‐dependent fluorescence enables accurate pH measurements by confocal microscopy and high‐throughput detection in sorting experiments. Droplets were imaged using a confocal microscope (Leica TCS SP8) equipped with a 10×/0.32 dry objective. A sequential excitation protocol was used to measure the ratiometric fluorescence of the droplets: HPTS was excited at 405 and 488 nm in two separate frames, while the emission was collected between 515 and 535 nm. This approach allowed us to maximize the accuracy of the inferred pH data. The local pH in each droplet was calculated based on the ratio of HPTS fluorescence intensities at the two excitation wavelengths. The pH of active droplets was compared to neighboring droplets to assess local diffusion effects (Figure S3).

We note that the phosphate buffer system used in this study comprises a mixture of monobasic sodium phosphate (NaH_2_PO_4_) and dibasic sodium phosphate (Na_2_HPO_4_). The buffering capacity of this mixture is strongest around pH 7.0–7.5, near the acid dissociation constant, pKa ≈ 7.2, of phosphoric acid. Because the pH monitoring and the microbial screening experiments are performed in calcium‐free medium, the phosphate ions are expected to remain stable in solution. In cultures containing metal ions that form precipitates with phosphates or in systems where phosphates might affect cell metabolism, one may consider the use of other biocompatible buffers, such as Tris, HEPES or MOPS.

### 4.10 Sorting of Urease‐Expressing Bacteria From Mock Consortium

RFP‐expressing ureolytic bacteria and CFP‐expressing control bacteria were encapsulated in agarose beads with loading densities of λ = 0.05 and λ = 0.2, respectively. The beads were incubated at 37°C for 15 h, after which the emulsion was broken, and the beads were filtered. A mock consortium was then prepared by mixing the two bead suspensions in a 1:20 ratio, resulting in a mixture of 0.5% RFP‐positive beads, 10% CFP‐positive beads, and 89.5% empty beads. The beads were re‐encapsulated into single emulsion droplets containing 0.5 _m_ urea, 50 mM sodium phosphate buffer, and 0.5 mM HPTS as a pH‐sensitive indicator. Droplets were sorted on a microfluidic platform using a voltage threshold of 0.6 V. Sorting enabled the separation of 628 out of approximately 240,000 droplets within 20 min. After sorting, the droplets were de‐emulsified, and the collected beads were analyzed using confocal microscopy and classified based on their fluorescence. CFP‐positive beads were identified as controls, while RFP‐positive beads indicated ureolytic activity.

### 4.11 Sorting of Ureolytic Bacteria From Soil Microbiome

Soil was collected from a forest near ETH Hönggerberg (47.4065°N, 8.5133°E) to test the applicability of the CiBiD assay for the bioprospection of urease‐active bacteria from natural microbial communities. For pre‐enrichment, 2.5 g of soil were dispersed in 10 mL of enrichment medium (EM) containing 0.25 g L^−^^1^ glucose, 13.8 g L^−^^1^ sodium acetate, 0.5 g L^−^^1^ peptone, and 20 g L^−^^1^ urea, following a previously reported formulation [48]. The mixture was incubated for 16 h at 30°C under shaking at 160 rpm. The culture was sonicated for 1 min to detach bacteria from soil particles and left to sediment for 30 min. The supernatant containing planktonic cells was filtered through a 5 µm mesh to remove debris and large aggregates. The filtered suspension was diluted 1:10 in fresh, nutrient‐rich enrichment medium containing 2.5 g L^−^^1^ glucose, 13.8 g L^−^^1^ sodium acetate, 10 g L^−^^1^ peptone, 20 g L^−^^1^ urea, and 0.1 M Tris buffer (pH 7.5), followed by encapsulation in 1.5 wt% low‐melting agarose beads as described above. The beads suspended in the oil phase were incubated at 30°C for 24 h to allow the formation of bacterial microcolonies. After solidification and emulsion breaking, the beads were collected and re‐encapsulated into single emulsion droplets containing 0.5 m urea, 40 mM sodium phosphate buffer (pH 7.5), and 0.5 mM HPTS as pH‐sensitive fluorescent dye.

The droplets were introduced into the microfluidic sorting device at an event rate of approximately 100 droplets s^−^^1^. Within 20 min, ∼80,000 droplets were analyzed, and 265 droplets surpassed the sorting threshold of 1.65 V. The sorted droplets and the waste fraction were de‐emulsified, and the recovered beads were plated on urea–peptone agar plates containing 10 g L^−^^1^ peptone, 10 g L^−^^1^ urea and 10 g L^−^^1^ agar, followed by incubation for 48 h at 30°C. From each condition, sixteen colonies were isolated directly from the agar plates for further analysis. Control isolates were obtained in parallel by directly plating serial dilutions of the soil suspension onto urea–peptone agar plates without prior encapsulation or microfluidic sorting. The colonies were picked randomly among all candidates that were well‐isolated and not touching any neighboring colonies. Microscopy imaging of the isolates revealed that a single agarose bead was located at the centre of the colony, indicating that each colony originated from a bead‐encapsulated microcolony rather than from an escaped cell originating from another droplet. Isolates were grown in liquid urea–peptone–TRIS medium (20 g L^−^^1^ urea, 10 g L^−^^1^ peptone, 0.1 M TRIS, pH 7.5) to an optical density at 600 nm (OD_600_) above 0.5, diluted to OD_600_ = 0.2, and plated on urea broth agar (Sigma Aldrich) to visualize urease activity. After 8 h of incubation at 30°C, ureolytic colonies were identified by the formation of a pink halo around the colony.

To quantify bulk urease activity, cultures were diluted 1:10 in 0.1 M HEPES buffer containing 0.5 M urea and 0.15 mg mL^−^^1^ phenol red as a pH indicator. Absorbance‐based pH measurements were performed in a microplate reader, and the pH values were calculated using calibration curves as described above.

## Conflicts of Interest

The authors declare no conflict of interest.

## Supporting information




**Supporting File 1**: smll72857‐sup‐0001‐SuppMat.docx.

**Supporting file 2**: smll72857‐sup‐0002‐VideoS1.avi.

## Data Availability

The data that support the findings of this study are available from the corresponding author upon reasonable request.
