# Supplementary material for: Cell‐in‐Bead‐in‐Droplet Platform for pH‐Based Microfluidic Screening of Ureolytic Bacteria
Source: Small. 2026 Feb 18;22(22):e08107. doi: 10.1002/smll.202508107 (PMC13089103; doi:10.1002/smll.202508107)
Supplement: Supplementary file 1 — Supporting File 1: smll72857‐sup‐0001‐SuppMat.docx. [file SMLL-22-e08107-s001.docx]

**Supplementary information**

**Bead permeability to nutrients**

The assess if agarose beads are sufficiently permeable to nutrients, we compare the incubation timescale with the diffusion timescale expected for nutrient molecules across the bead. The diffusion timescale is given by $L^{2}/D$, where $L$ is the diffusion length scale and $D$ is the diffusion coefficient of the molecule through the medium (gel). For 2wt% agarose gels, one should expect the diffusion coefficient of molecules to depend on the hydrodynamic radius ($r$) of the diffusing molecules. Under the reasonable assumption that the molecules of the culture medium show $r$ below 10nm, one should expect their diffusion coefficient in the agarose gel to be approximately 0.6$D_{0}$, [1] where $D_{0}$ is the diffusion coefficient in solution. Assuming a molecular diffusion coefficient ($D_{0}$) in water of ∼10^−10^ m^2^s^−1^, it should take less than 7 seconds for the nutrients of the medium to diffuse through a bead with a radius of 20$\mu$m ($L$). This diffusion timescale is many orders of magnitude lower than the incubation time (4-24h). Such analysis indicates that the diffusion of nutrients should not be a limiting factor for the growth of the microorganisms in beads.

**Plasmid construction and urease expression**

*Plasmid Design and Construction*

To enable fluorescent labeling and inducible urease expression in *E. coli*, a set of custom plasmids was constructed and characterized. These plasmids served two primary purposes: first, to provide constitutive fluorescence for tracking and sorting; and second, to express the urease operon from *Sporosarcina pasteurii* under inducible control. Plasmid pKTT [2] contains a pSB4K5 backbone, and constitutively expressed mTurquoise2 cyan fluorescent protein. Plasmids pLUO4-GFP and pLUO4-RFP contained constitutively expressed fluorescent reporters as well inducible urease expression (Figure S4). While GFP-expressing strains were used during initial optimization experiments, all data presented in this study were obtained using the RFP-expressing strain. These were built on backbone of plasmid pL6FO [3] which contained a pSC101 origin of replication and a kanamycin resistance gene. The plasmid was further engineered to contain a constitutively expressed fluorescent reporter, either superfolder GFP or mCherry RFP. A further IPTG inducible system, containing constitutively expressed LacI repressor and the pL-lac promoter [4] expressed the ureABCEFDG urease operon from *Sporosarcina pasteurii* downstream of a riboJ insulator [5]. In the presence of nickel ions, and IPTG (Isopropyl β-d-1-thiogalactopyranoside), *E. coli* bearing these plasmids are expected to be urease positive. Physical DNA and full sequence information of pLUO4-GFP/RFP can be found at Addgene (plasmid #TODO).

DNA construction was performed using Gibson assembly, [6] following the protocol in reference using enzymes Taq ligase (M0208S), T5 Exonuclease (M0663S), and Phusion polymerase (M0530S), obtained from New England Biolabs (NEB). PCR fragments were obtained using custom DNA oligos (Integrated DNA Technologies) on the templates specified, designed to contain 25-30 bp overlaps between assembled fragments. The urease operon DNA was obtained through PCR amplification using *S. pasteurii* DSM33 genome as template, using primers ureOP_sUTR_F: tccgtgaggacgaaacagcctctacaaattttgtttaaTTCGAGTTAAGTGTAAAGGAGG and UreOp_R: ggaggcctcttttctggaatttggtaccgagTTACAATAGGTGCACCATTATAAAATCTC. In these primers, capitalized sequence is homologous to the *S. pasteurii* genome, whereas the lowercase sequence represents overlaps to the designed plasmid for assembly. PCR was performed using the Q5 ® Hot Start High-Fidelity (M0494S) from NEB in a Thermocycler machine (MiniAmp, Applied Biosystems), following the standard Q5 protocol from the manufacturer. PCR fragments were purified with Reliaprep DNA Clean-Up and concentration system (A2892, Promega). Cloning was performed in *E. coli* DH5a chemically competent cells. Plasmid purification was performed with the PureYield Plasmid Miniprep System (A1223, Promega). Plasmid DNA sequence was confirmed by Sanger sequencing carried out by MicroSynth, Switzerland.

*Urease activity tests*

Urease activity of *E. coli* DH5a harbouring plasmids pLUO4-GFP or pLUO4-RFP was characterized via changes in pH detected by monitoring the absorbance signal of 15 mg/mL Phenol Red (PR) dye. PR is yellow in acidic conditions, and transitions to pink/purple at higher pH, with a peak in absorbance around 560 nm arising at basic conditions. For characterization, plasmid-bearing *E. coli* were initially grown in LB medium with 50 µg/mL kanamycin overnight at 37°C under shaking conditions. These cultures were then used 1:1000 to inoculate fresh LB medium, supplemented with 50 µg/ml kanamycin, 15 µg/mL PR, 50 µM NiCl_2_, 0.5 M urea, and variable amounts of IPTG inducer. Inoculated cultures were grown in 200 µL black 96-well plates (F-bottom, µClear, Greiner Bio One) covered with an impermeable film inside a microplate reader (Varioskan Lux, Thermo Scientific) by incubation at 37°C with a continuous high shaking between reads for 15 hours. Every 10 mins, absorbance at 435, 560, 650 nm, and GFP (Ex 485 nm, Em 520 nm) and RFP (Ex 584 nm, Em 610 nm) fluorescence were measured. Cell density was tracked by absorbance at 650 nm (A650), as PR shows no absorbance at this wavelength [7]. pH was monitored by measuring the absorbance at 560nm (A560), which was initially calibrated with 15 µg/mL PR in LB medium that had been pH adjusted to values in the range 3-10. The A560 values showed a sigmoidal correspondence to pH that could be closely fitted by a 3-parameter logistic equation. To determine the pH of bacterial cultures, absorbance due to cells was removed by subtracting A650. The resulting corrected A560 values were converted to pH, showing that cultures with at least 100 µM IPTG had increased pH to over 9 through the breakdown of urea (Figure S4 B,C,H). The increase in pH is accompanied by weaker cell growth and fluorescent protein expression, as *E. coli* grow poorly in such high pH conditions (Figure S4 D-G). After 48 hours, all *E. coli* + pLUO4-GFP/RFP wells had turned pink, unlike a media control, indicating basal expression during stationary phase is enough to generate significant urease activity and raise pH (Figure S4 I).

**Fabrication of Microfluidic Devices**

Microfluidic devices were fabricated using standard photolithography methods by patterning the photoresist SU-8 (3000 series, MicroChem) on silicon wafers to create masters for soft lithography. Briefly, a 1:10 mix ratio of polydimethylsiloxane (PDMS, Sylgard™ 184, Dow Corning) was mixed and poured onto the silicon master, degassed, and cured at 70°C for 8 hours. Once cured, inlet and outlet holes were punched and the microfluidic devices bonded using air-plasma treatment.

Microfluidic devices for emulsification were bonded to glass slides (FisherbrandTH Superfrost™) following a 20-second air-plasma treatment at 4×10⁻¹ mbar on medium level (Plasma Cleaner PDC-32G) and then placed on a hot plate at 70°C for 1 hour to enhance bonding. Microfluidic devices for sorting were bonded to PDMS-coated silicon wafers (spin-coated at 2,400 rpm for 10 seconds and cured) using a 1-minute air-plasma treatment (Zepto, Diener), heated on a hot plate at 120°C for 2 hours, and subsequently cut and bonded to 50 × 24 × 0.17 mm glass coverslips (Glaswarenfabrik Karl Hecht) using the same air-plasma treatment.

The channels of microfluidic devices were hydrophobized using a 1 vol% solution of 1H,1H,2H,2H-perfluorooctyltrichlorosilane (Fluorochem) in HFE-7500 (3M). For sorting devices, electrodes were fabricated by inserting low melting point solder (51In/32.5Bi/16.5Sn; Indium Corporation) at one end of the electrode channel and a wire at the other end. The entire chip was placed on a hot plate at 150°C for 3 minutes to melt and reflow the solder across the channel.

**Encapsulation of fluorescent particles in agarose beads**

Fluoro-Max particles (1 μm, 1% solid loading, Thermo Scientific) were incorporated at 3 wt% into a 1.5 wt% melted agarose solution before encapsulation. Hydrogel beads containing fluorescent particles were generated and subsequently emulsified into a second emulsion using flow-focusing microfluidics. Bead concentrations were adjusted to achieve mean number of beads per droplet (*λ*) values of 0.1 and 0.5. Confocal microscopy was used to capture images and measure the size of the agarose beads, the size of the single emulsion droplets, and the occupancy rate of beads within the droplets.

**Encapsulation of *E. coli* expressing RFP and urease in agarose beads**

An overnight culture of *E. coli* in the exponential growth phase (OD 600 between 0.8 and 1.0) was resuspended in fresh LB medium containing 50 µM NiCl₂ and 100 µM IPTG, and subsequently encapsulated within agarose beads. To determine the optimal cell concentration for achieving single-cell occupancy (λ = 0.1), the initial culture was diluted at ratios of 1:10, 1:50, 1:100, 1:200, and 1:500. Droplet occupancy rates were assessed by counting the number of empty and occupied droplets in confocal microscopy images.

**Supplementary table**

**Table S1**. Operational parameters used for the microfluidic bead generation, droplet generation and droplet sorting processes described in this study.

| **Device / Step** | **Parameter** | **Value** | **Notes** |
| --- | --- | --- | --- |
| **Bead**  **generation** | Continuous phase | HFE-7500 + 2 wt% fluorosurfactant | Oil used for droplet formation |
|  | Dispersed phase | Cell suspension in 1.5 wt% agarose | Agarose prepared in milliQ or growth medium |
|  | Aqueous inlet pressure | 100 mbar (≈ 500 µL/h) | Elveflow pressure controller |
|  | Oil inlet flow rate | 500 µL/h | Harvard Syringe Pump |
|  | Temperature during encapsulation | 37 °C | Maintains agarose in liquid state |
| **Droplet**  **generation** | Continuous phase | HFE-7500 + 2 wt% fluorosurfactant | Same formulation as bead generation |
|  | Dispersed phase | Bead suspension in assay buffer | Contains urea, buffer, pH indicator |
|  | Aqueous inlet flow rate | 750 µL/h | Harvard Syringe Pump |
|  | Oil inlet flow rate | 3 mL/h | Harvard Syringe Pump |
| **FADS sorting** | Detection wavelength / filter set | Excitation 488 nm; Emission 520/35 nm; 30 μm pinhole | 488 nm solid-state laser (Omicron) |
|  | Photodetector gain / PMT voltage | Hamamatsu H10722-20; 0.7 kV | Gain not explicitly measured |
|  | Fluorescence threshold | User-defined | Set in LabVIEW interface |
|  | Sorting mode | Sort above threshold | High-fluorescence droplets triggered |
|  | Sorting frequency | ~200 droplets/s |  |
|  | Sample flow at nozzle | 120 mbar | Fluigent LineUP |
|  | Sheath oil flow | 8 µL/min + 8 µL/min | Produces droplet spacing |
|  | Sort window width | 0.6–0.8 ms | 25–35 pulses @ 40 kHz |
|  | Detection sampling rate | 100 kHz | NI PXI-7842R FPGA |

**Supplementary figures**


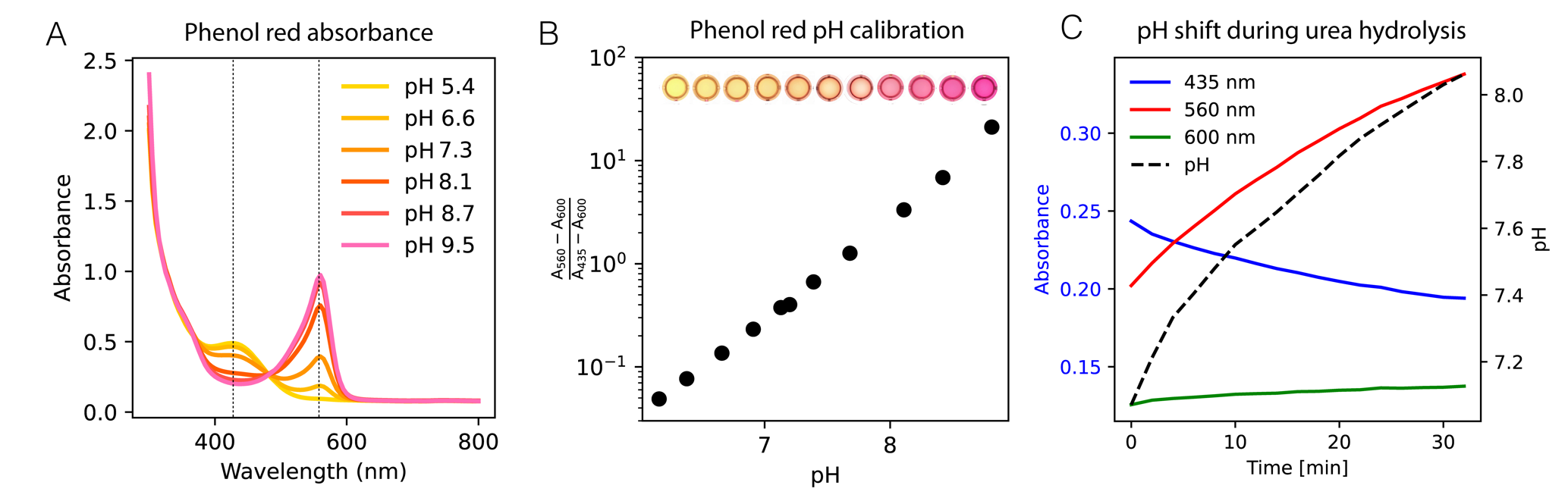


**Figure S1: Monitoring of bulk urea hydrolysis in plate reader using phenol red as a pH indicator.** (A) Absorbance spectra of phenol red (0.05 mg/mL) at pH values ranging from 5.4 to 9.5, showing characteristic pH-dependent changes in absorbance near 435 nm and 560 nm. (B) Calibration curve constructed by plotting the absorbance ratio $\frac{A_{560}\text{-}A_{600}}{A_{435}\text{-}A_{600}}$ against pH. The inset shows the colors of the corresponding wells of the plate reader, illustrating the visual transition of phenol red. (C) Time-resolved absorbance measurements during urea hydrolysis by urease-expressing, cell-laden agarose beads dispersed in an aqueous solution containing urea, sodium phosphate buffer, and phenol red. The pH increase (black dashed line) can be quantitatively measured from the spectral shift in phenol red absorbance, enabling non-invasive tracking of enzymatic activity in bulk.


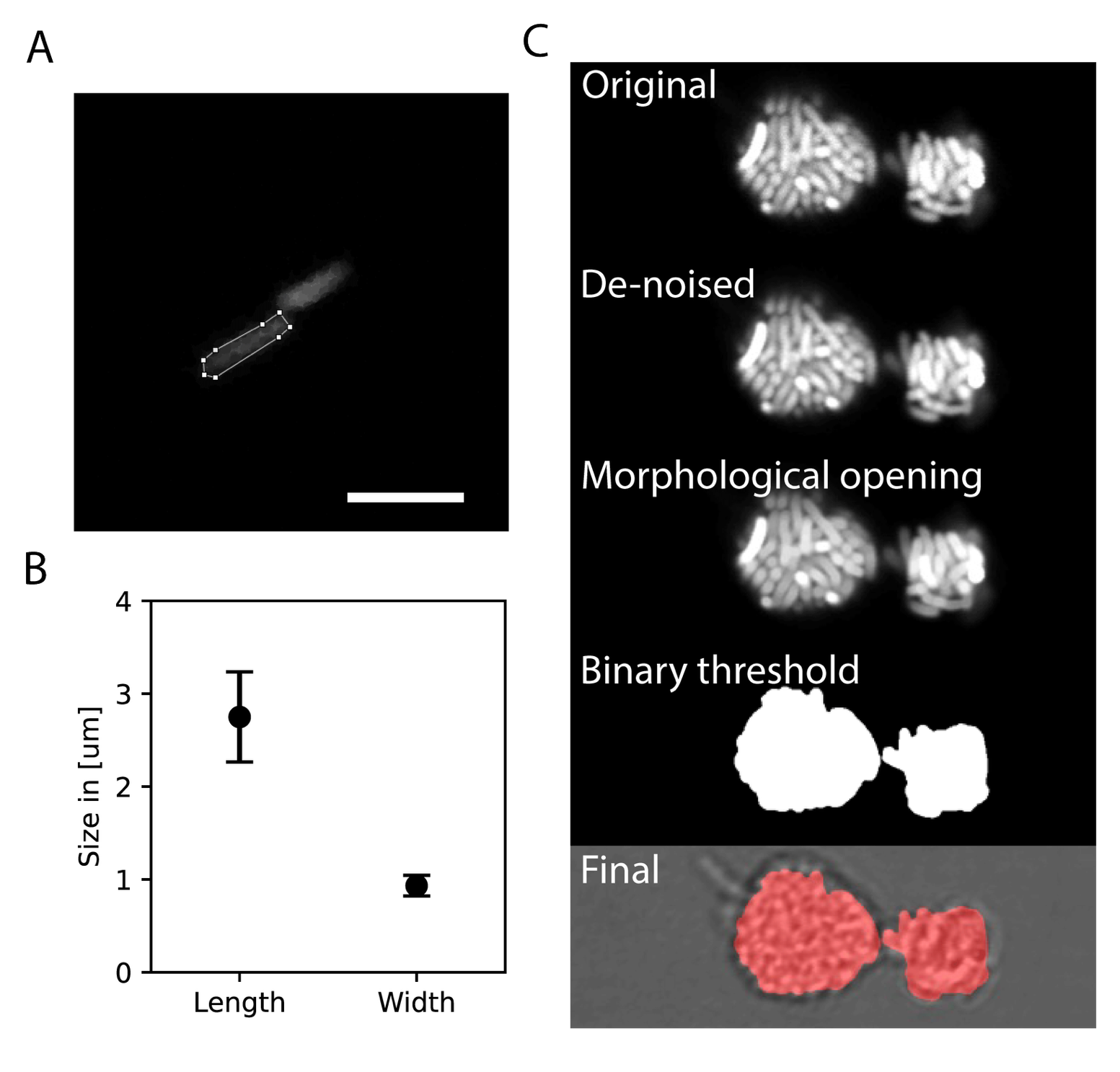


**Figure S2: Quantification of cell count within cell-laden hydrogel beads.** (A) Representative fluorescence image of a single bacterial cell expressing red fluorescent protein (RFP), with a fitted bounding box used to estimate cell dimensions. Scale bar: 5 µm. (B) Quantification of single-cell dimensions (n > 50), with error bars representing standard deviation. The volume of an individual bacterium was approximated as V_cell_ = Length * Width^2^. (C) Image processing pipeline for colony segmentation from a single slice of a z-stack (z-step = 1 µm). Top to bottom: original fluorescence image, de-noised image, morphological opening to reduce background and separate clustered objects, binary thresholding for mask generation, and final segmented colonies overlaid on the original image. This segmentation process allows for the calculation of the total colony volume. By dividing the colony volume by the average single-cell volume, an estimate of the number of cells per colony can be obtained.


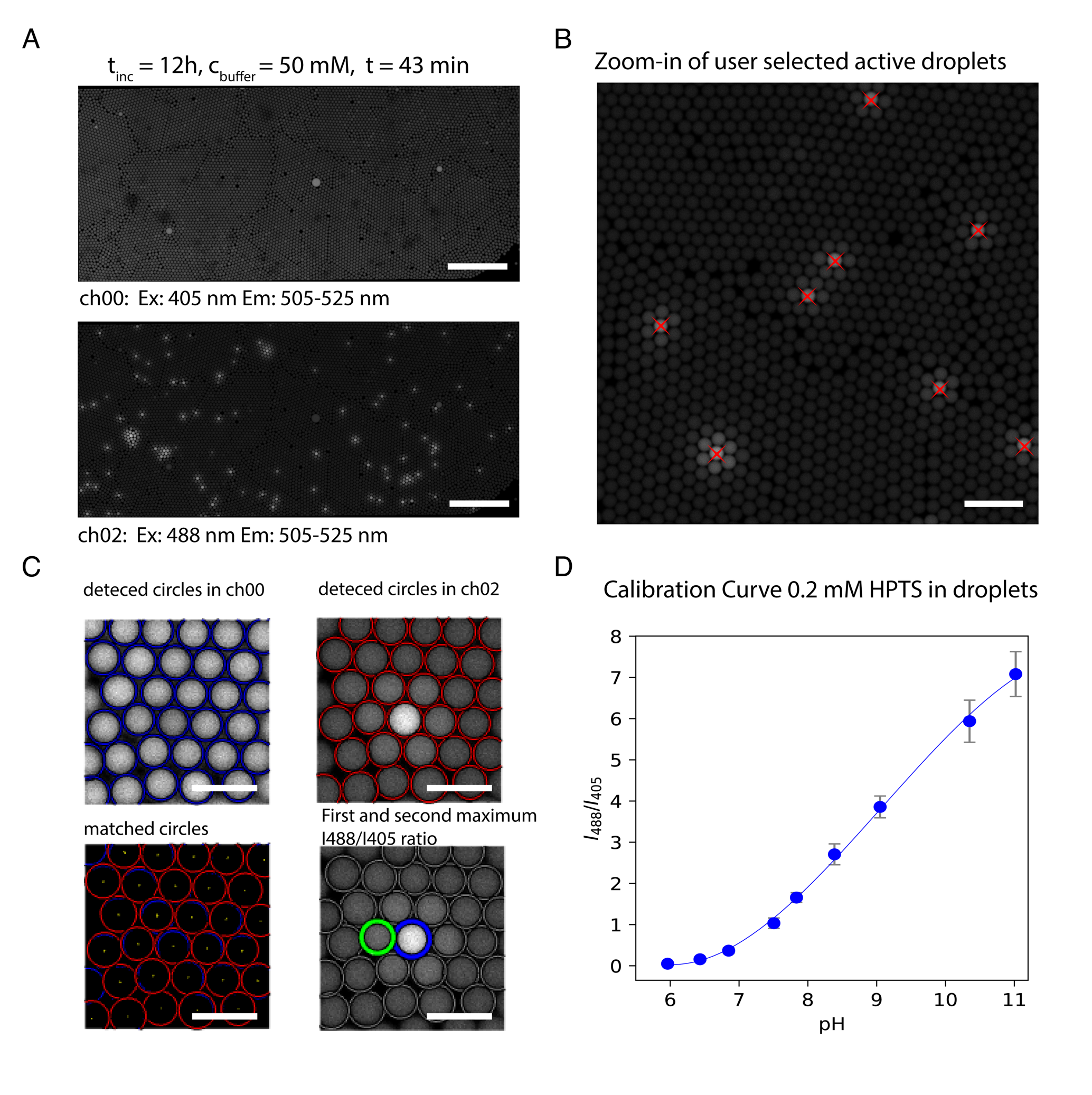


**Figure S3: Automated image analysis pipeline for single-droplet pH quantification using HPTS as ratiometric fluorescenct dye.** (A) Representative confocal microscopy images of droplets containing 0.2 mM HPTS at 43 minutes after re-encapsulation. Two fluorescence channels were recorded: ch00 (I405) with excitation of 405 nm and emission wavelength of 505–525 nm; and ch02 (I488) with excitation of 488 nm and emission wavelength of 505–525 nm. Scale bars correspond to 1 mm. (B) Zoom-in on user-selected droplets with high fluorescence in ch02, indicating active cell-laden droplets. Scale bars correspond to 200 μm. (C) Droplet detection and matching pipeline: circles are independently detected in ch00 (blue) and ch02 (red) and matched to quantify the I488/I405 ratio per droplet. The droplets with the highest (cyan) and second-highest (green) ratios are highlighted. Scale bars correspond to 100 μm. (D) Calibration curve of the fluorescence intensity ratio I488/I405 as a function of pH in droplets containing 0.2 mM HPTS, showing strong sensitivity in the pH range 7 –10. Error bars represent standard deviation from three replicate measurements.


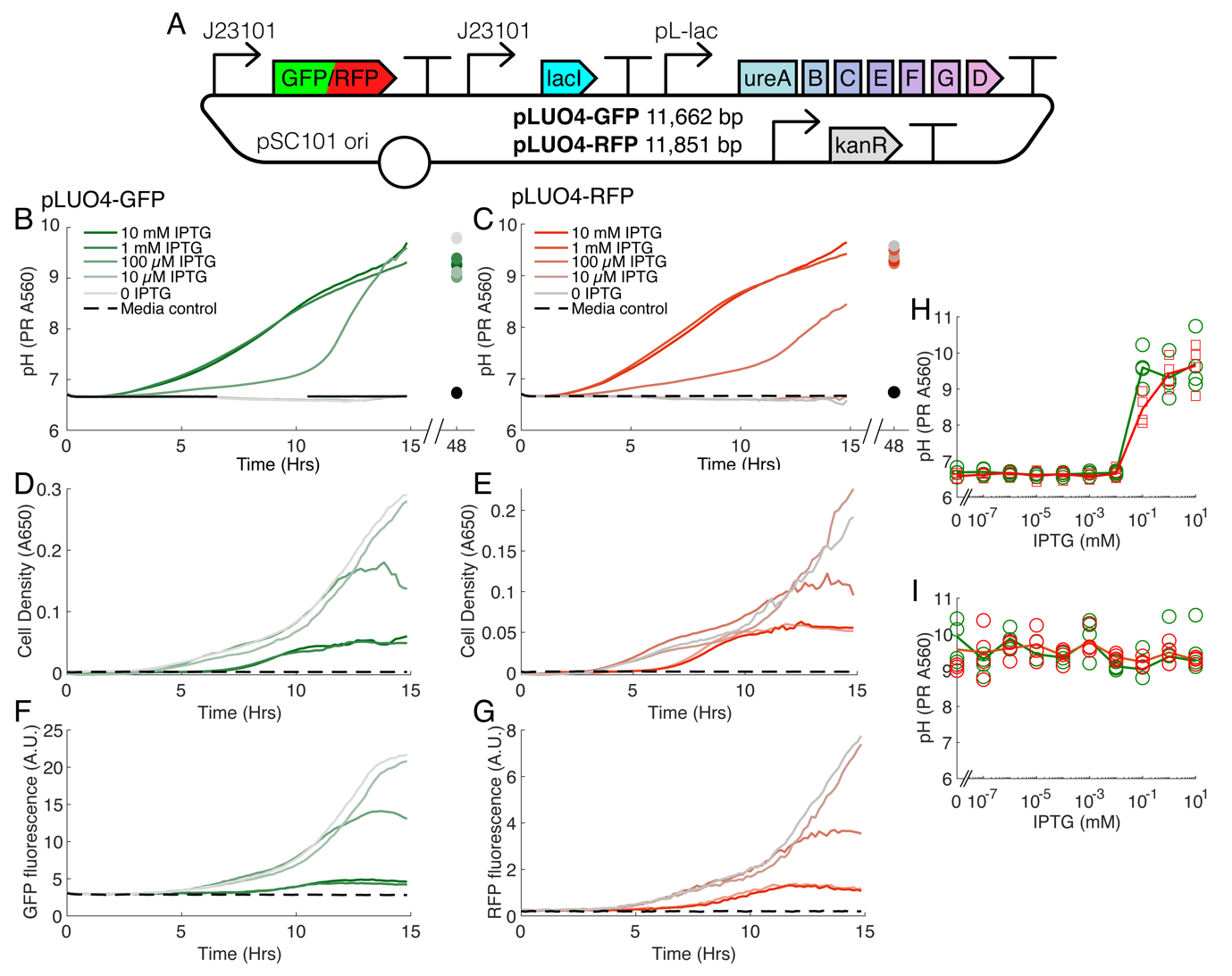


**Figure S4: Plasmid construction and activity of engineered *E. coli*.** (A) Plasmid map for pLUO4-GFP and pLUO4-RFP. *E. coli* harbouring these plasmids showed IPTG dependent urease activity when grown in LB media, increasing (B,C) pH through the breakdown of urea into ammonia. (D,E) Bacterial growth and (F, G) fluorescent reporter activity was comparable in both strains, with IPTG-induced pH increases harming growth and reporter gene expression. Lines in time-course data represent average values of 4 replicates. Culture pH at (H) 15 hours was neutral like controls at IPTG concentrations below 100 µM, but increased to >9 at higher concentrations. (I) After 48 hours at room temperature, these same bacterial cultures had increased pH, irrespective of IPTG concentration. Here red and green lines show average pH of cultures with pLUO4-GFP and -RFP,respectively, and circles and squares represent individual data points.

**Supplementary video**

[**Time-lapse confocal microscopy imaging of *E. coli* colony growth in agarose beads**](https://polybox.ethz.ch/index.php/s/CRKJNr395KP3ptL)

[Click here](https://polybox.ethz.ch/index.php/s/CRKJNr395KP3ptL)

*E. coli* expressing GFP were encapsulated in agarose beads and incubated in droplets at 37 °C. Time-lapse confocal microscopy reveals bacterial growth over time, with colonies beginning to expand after 3 hours of incubation. As colonies grow, some reach the oil–water interface and start colonizing the droplet surface, indicating a potential route for bacterial cell loss during de-emulsification. Images were captured for 10 hours overnight using a confocal microscope in a heated chamber. Scale bar: 50 µm.

**References**

1. Pluen, A.; Netti, P. A.; Jain, R. K.; Berk, D. A., *Biophys. J.* **1999,** *77* (1), 542–552. DOI <https://doi.org/10.1016/S0006-3495(99)76911-0>.

2. Rudge, T. J.; Federici, F.; Steiner, P. J.; Kan, A.; Haseloff, J., *ACS Synthetic Biology* **2013,** *2* (12), 705–714. DOI 10.1021/sb400030p.

3. Kan, A.; Joshi, N. S., *MRS Communications* **2019,** *9* (2), 441–455. DOI 10.1557/mrc.2019.28.

4. Lutz, R., *Nucleic Acids Res.* **1997,** *25* (6), 1203–1210. DOI 10.1093/nar/25.6.1203.

5. Lou, C.; Stanton, B.; Chen, Y.-J.; Munsky, B.; Voigt, C. A., *Nat. Biotechnol.* **2012,** *30* (11), 1137–1142. DOI 10.1038/nbt.2401.

6. Gibson, D. G.; Benders, G. A.; Axelrod, K. C.; Zaveri, J.; Algire, M. A.; Moodie, M.; Montague, M. G.; Venter, J. C.; Smith, H. O.; Hutchison, C. A., *Proceedings of the National Academy of Sciences* **2008,** *105* (51), 20404–20409. DOI 10.1073/pnas.0811011106.

7. Elmas, S.; Pospisilova, A.; Sekulska, A. A.; Vasilev, V.; Nann, T.; Thornton, S.; Priest, C., *Sensors* **2020,** *20* (11), 3099. DOI 10.3390/s20113099.
